# Linear and branched polymer prodrugs of the water-soluble nucleoside reverse-transcriptase inhibitor emtricitabine as structural materials for long-acting implants†

**DOI:** 10.1039/d2tb00825d

**Published:** 2022-05-19

**Authors:** Anika Shakil, Faye Y. Hern, Chung Liu, Kartik Temburnikar, Pierre Chambon, Neill Liptrott, Tom O. McDonald, Megan Neary, Andrew Owen, Caren Freel Meyers, Steve P. Rannard

**Affiliations:** Department of Chemistry, University of Liverpool Crown Street Liverpool L69 7ZD UK srannard@liverpool.ac.uk; Materials Innovation Factory, University of Liverpool Crown Street L69 7ZD UK; Centre of Excellence in Long-acting Therapeutics (CELT), University of Liverpool Liverpool L7 3NY UK; Department of Pharmacology and Molecular Sciences, The Johns Hopkins University School of Medicine 725 North Wolfe St. Baltimore MD, 21205 USA; Department of Pharmacology and Therapeutics, Institute of Systems, Molecular and Integrative Biology, University of Liverpool Liverpool L7 3NY UK

## Abstract

Long-acting drug delivery is a growing area of interest as it overcomes many challenges related to patient adherence to therapy and the pill burden associated with chronic illness. Injectable formulations are becoming more common and drug-releasing implants also provide several opportunities. Highly water soluble drug compounds are poor candidates for long-acting delivery. Here, the water-soluble nucleoside reverse transcriptase inhibitor emtricitabine (FTC) has been used as a novel A–B monomer in step-growth polymerisation with chloroformate functional C_*n*_ monomers, to produce new poly(carbamate/carbonate) structures with varying architecture. The polymer prodrugs were all solid at ambient temperature and have been shown to release FTC when subjected to mixed gender human plasma. Vacuum compression moulding has been used to form solid rod implants without polymer degradation; the rods show FTC release over long periods in the presence of microsomes, establishing the basis of a polymer prodrug strategy for FTC delivery.

## Abstract 2

Long acting release of the water soluble nucleoside reverse transcriptase inhibitor emtricitabine (FTC) has been demonstrated *in vitro* through polymer formation using FTC as a monomer, and forming implants made of the drug containing polymer.

## Graphical abstract

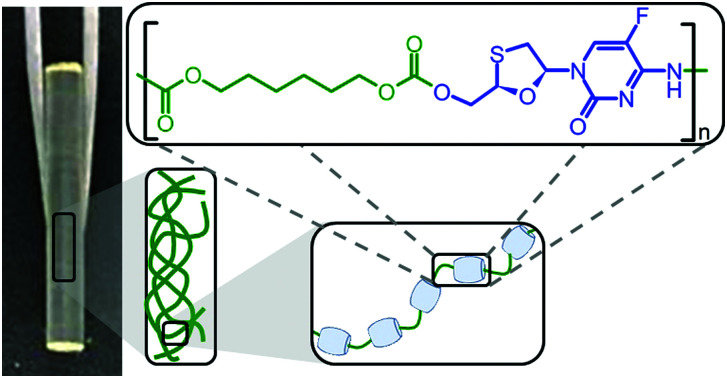

## Introduction

In recent years there has been an increasing focus on the development and clinical evaluation of long-acting therapeutics. Strategies that can enable the maintenance of drug exposure within the therapeutic window for weeks to months after a single administration may have considerable clinical benefits for acute and chronic diseases alike. Daily oral dosing is the foundation for many therapies. However, it is widely accepted that patient adherence to daily dosing is challenging even over durations as short as one week. Poor adherence has a number of implications including compromised efficacy, emergence of resistance and significant cost.^1^ For conditions requiring prophylaxis or therapy to maintain health, a commitment to therapy may be required for many years or even an entire lifetime. Well documented examples include up to 50% of patients showing poor adherence to daily statins,^2^ poor clinical outcomes and growing antibiotic resistance from oral dosing,^3^ and low viral suppression and multi-drug resistance in poorly adherent HIV-infected patients.^4^

For those living with HIV, daily antiretroviral (ARV) combination therapies are essential to maintain a low viral load, prevent transmission and enable a near-normal life expectancy.^5^ Up to three ARVs are typically used with regimens selected for each patient dependent on their history of ARV medication.^6^ Nucleoside reverse transcriptase inhibitors (NRTIs) are considered the “backbone” of ARV therapies and are typically administered as a two-drug combination in addition to a third ARV from a different class.^7^ NRTIs are generally water soluble-drug compounds whilst other ARVs may be highly insoluble. The burden of lifelong pill taking can lead to poor adherence and subsequent consequences that can severely impact quality of life. To attempt to alleviate the burden, several fixed dose combination (FDC) pills are now clinically available, offering up to three drugs within a single dose. Although FDCs aid the simplification of ARV therapy and reduce the risk of incorrect dosing,^8^ with subsequent improvement in adherence,^9^ the patient is still the primary determinant for the daily management of their condition.

**Fig. 1:**
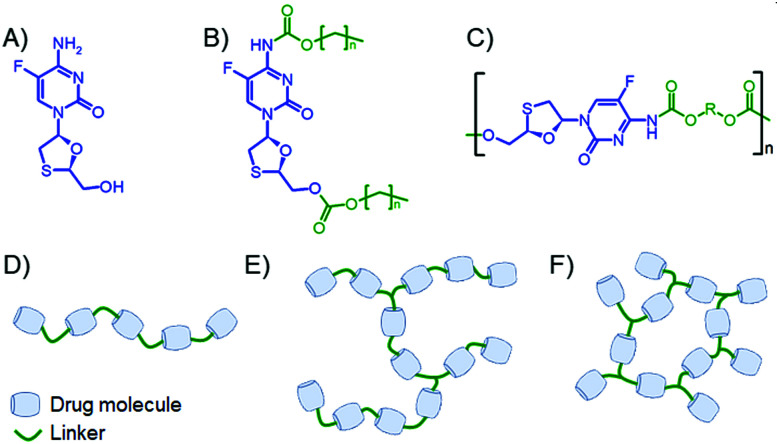
Schematic representation of the strategy for polymers containing emtricitabine (FTC) in the backbone. (A) FTC; (B) small molecule disubstituted carbonate/carbamate prodrugs; (C) targeted FTC-containing polymers as the subject of this study; (D) linear polymer target; (E) lightly branched polymer target; and (F) crosslinked polymer target.

Long-acting therapeutics have been available for many years for maintenance of chronic, and treatment of acute, schizophrenia. Paliperidone palmitate received US Food and Drug Administration approval in July 2006 as an intramuscular long-acting injectable (LAI) as an alternative to an oil depot or oral antipsychotic drugs. By increasing the hydrophobicity of paliperidone through the formation of the palmitate prodrug, the delivery of the parent paliperidone was controlled so that a single injection was able to replace daily oral dosing for up to three months with significant patient benefits,^10^ with recent approvals for a six monthly injection.^11^ The potential for LAI interventions for HIV^12^ has also recently been realised through the FDA approval of a bimonthly combination of two injectable ARVs, cabotegravir and rilpivirine, known as Cabenuva; additional approvals were also achieved in the UK, Canada and Europe.^13^ Cabotegravir is an integrase inhibitor and rilpivirine is a non-nucleoside reverse transcription inhibitor and Cabenuva not only represents the first LAI for HIV, but it is also the first widely available complete regimen that does not utilise an NRTI backbone.^14^ The water solubility of NRTIs presents a considerable hurdle for their application in LA medicines; however, recently we described the formulation of stable lipid nanoparticles^15^ after hydrophobic modification of the NRTI lamivudine. Furthermore, we have also demonstrated the formation of semi-solid hydrophobic prodrug nanoparticle formulations of the NRTI emtricitabine (FTC), Fig. 1A, that were slowly activated by muscle and plasma enzymes to deliver the parent NRTI over prolonged periods.^16^ The small molecule prodrugs were formed by the one-pot reaction of alkyl chloroformates and the disubstitution of FTC at the primary amine and hydroxyl functional group, Fig. 1B. These carbamate/carbonate prodrugs displayed very low melting points but emulsion templated freeze drying^17–19^ allowed the creation of liquid dispersions that were readily syringeable.

The creation of long-acting options for water-soluble NRTIs offers the potential to pair with either cabotegravir or rilpivirine and produce new therapeutic combinations. Examples of long-acting drug delivery have utilised several strategies including implants, injectables^20,21^ and oral dosage forms,^22^ although the phenomenon of undigested “ghost” pills that fail to release drug has been reported clinically^23^ and many challenges exist for orally administered long-acting medicines.

Taking inspiration from hydrolytically cleaving polymer prodrugs^24–27^ that utilise multifunctional drugs as monomers, we present here a strategy for forming polymers using FTC as a backbone monomer of poly(carbamate–carbonate) polymers with differing architecture, Fig. 1C–F. The resulting polymers have been studied for activation and release of the parent FTC and their ability to form solid implants.

## Results and discussion

### Step-growth synthesis and characterisation of FTC-containing polymers

**Scheme 1:**
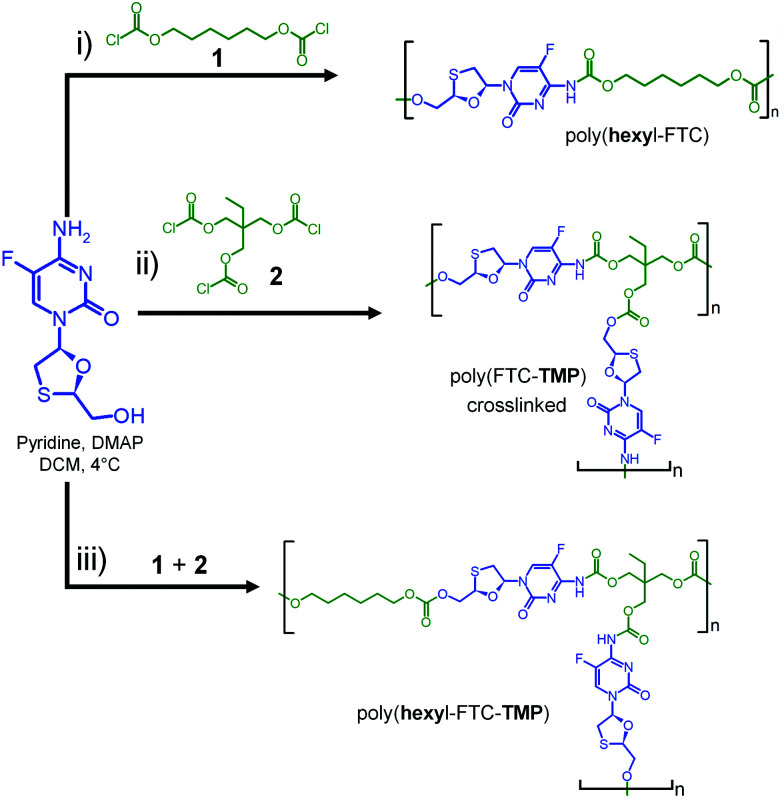
Synthesis strategy for FTC-containing carbamate/carbonate polymers with different architectures. (i) Linear, (ii) crosslinked, and (iii) branched polymer analogues.

Our previous studies of FTC prodrug synthesis focussed on the reaction of alkyl chloroformates, but the bifunctional nature of FTC allows the option of an A–B + C_*n*_ step growth polymerisation strategy by employing multifunctional chloroformate monomers. As the alkyl carbamate/carbonate substitution of FTC was shown to be cleavable under physiological conditions by naturally occurring and relevant enzymes, we hypothesised that the polymeric form of our earlier prodrugs would also be susceptible to enzymatic cleavage. Three polymerisation options were studied that offer variation in polymer architecture and allow an evaluation of the impact of architecture on the activation of the polymeric prodrugs with subsequent release of FTC, Scheme 1. Hexamethylene bis(chloroformate), 1, was selected as a C_2_ monomer to form linear polymers and the tris(chloroformate) of trimethylol propane (TMP), 2, was utilised as a C_3_ monomer to form branched and crosslinked materials; both materials were obtained *via* a custom synthesis (Fig. S1–S4, ESI†).

**Fig. 2:**
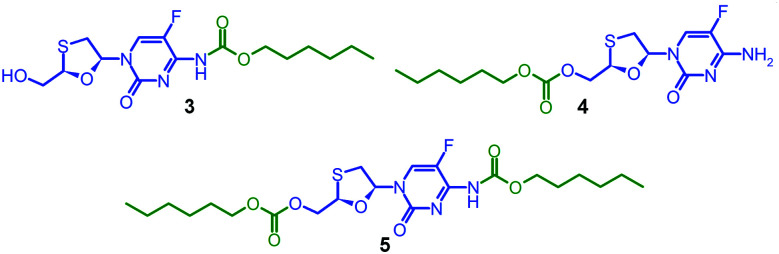
Model FTC compounds synthesised to enable polymer characterization.

Due to the expected analytical complexity of the FTC-containing polymers, a series of small molecule model compounds were synthesised to enable the assignment of ^1^H and ^13^C nuclear magnetic resonance (NMR) spectroscopy spectra. The chain ends of step-growth polymers derived from A_2_ + B_2_ polymerisations may contain either A functionality or groups that result from the presence of a B_2_ monomer residue at the terminal unit. Here, the polymers may have chain-ends comprising FTC primary amine and FTC primary hydroxyl, from the A–B monomer, in addition to primary hydroxyl groups from the hydrolysis of unreacted chloroformates derived from the C_*n*_ linkers. As such, hexyl chloroformate was reacted under differing reaction conditions to form either the monosubstituted carbamate derivative of FTC, 3, the monosubstituted carbonate derivative of FTC, 4, or the disubstituted carbamate/carbonate derivative, 5, Fig. 2. Through variation of base concentration within the reaction of hexyl chloroformate with FTC, the formation of 5 was achieved as a minor component of the crude reaction mixture while directing the major product to be either 3 or 4.

Column chromatography allowed the isolation of 3, 4 and 5 in high purity. Electrospray mass spectrometry (ESI-MS) identified 3 and 4 with identical adducts ([M + H]^+^ = 376.1 Da; [M + Na]^+^ = 398.1 Da) but a different fragmentation pattern strongly indicating formation or absence of the hexyl carbamate group (Fig. S5–S7, ESI†). The ESI-MS spectrum of 5 confirmed the disubstituted product ([M + H]^+^ = 504.2 Da; [M + Na]^+^ = 526.2 Da) and contained species that corresponded with the fragmentation pattern of 3. Fourier-transform infrared spectroscopy (Fig. S8–S10, ESI†) was conducted to provide a more complete characterisation and ^1^H and ^13^C NMR analyses were supplemented by heteronuclear single quantum coherence (HSQC) experiments to establish a clear basis for assignment (Fig. S11–S19, ESI†).

The synthesis of the linear poly(hexyl-FTC), Scheme 1, was achieved through the reaction of an equimolar ratio of FTC and hexamethylene bis(chloroformate), quenching of unreacted chloroformate with ethanol, and purification of the crude product by dilution with dichloromethane followed by liquid–liquid extraction prior to solvent removal. Poly(hexyl-FTC) was characterised using single detection oligomer size exclusion chromatography (SEC) using DMF as the eluent. It was clear that the polymerisation progressed as expected but produced a low molecular weight polymer with a polymethylmethacrylate (pMMA) equivalent number average molecular weight (*M*_n_) of 3170 g mol^−1^, weight average molecular weight (*M*_w_) of 4210 g mol^−1^ and a relatively narrow dispersity (*Đ*) of 1.46, Table 1, possibly as a result of extensive purification.

**Table 1:** Size exclusion chromatography (SEC) and differential scanning calorimetry (DSC) characterisation of linear, branched and crosslinked polymer samples containing FTC

Polymer	SEC^a^			FTC content (wt%)	DSC^c^
	*M*_n_ b (g mol^−1^)	*M*_w_ b (g mol^−1^)	*Đ*		*T*_g_ (°C)
poly(hexyl-FTC)	3070	4530	1.48	58.8	28
poly(hexyl-FTC-TMP)	4110	9800	2.38	57.7	34
poly(FTC-TMP)	Gel		—	63	51

a DMF eluent.

b Polymethyl methacrylate equivalent values.

c Values reported from the second heating cycle (heating rate of 5 °C min^−1^).

**Fig. 3:**
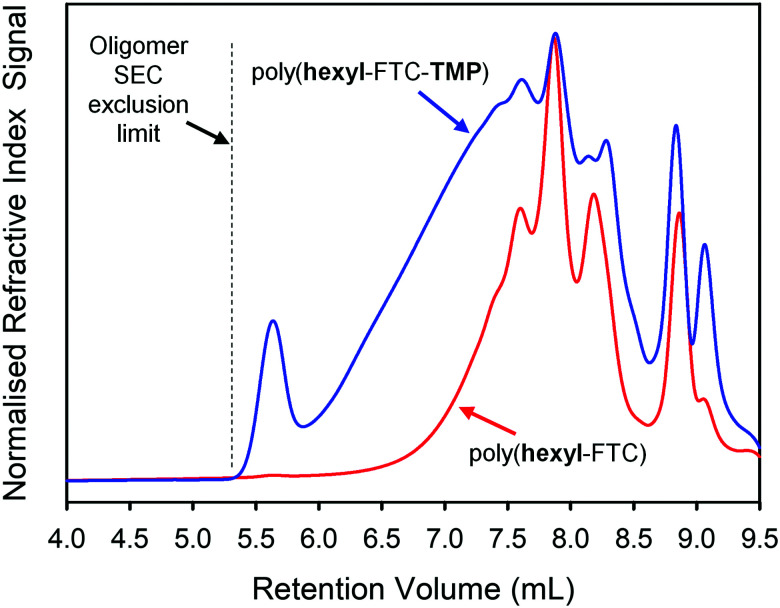
Oligomer size exclusion chromatography analysis (refractive index detection) and comparison of linear and branched FTC-containing polymers (DMF eluent).

As explained by the modified Carothers equation (eqn (S1), ESI†), step-growth polymerisation containing multifunctional monomers may lead to crosslinked polymers (as defined by a number average degree of polymerisation (DP_*n*_) of ∞). This occurs specifically if the average functionality of the monomers within the reaction is >2 as gelled polymers may be formed at relatively low monomer conversions. To provide non-crosslinked, soluble branched polymers containing FTC, we targeted a reaction of FTC with a mixture of 1 and 2 containing a 3 : 1 molar ratio. This generates an average monomer functionality of 2.125 and a theoretical gel point (DP_*n*_ = ∞) at approximately 94% conversion. The formation of poly(hexyl-FTC-TMP) was conducted over 24 hours and quenched with ethanol as conducted previously in the synthesis of poly(hexyl-FTC). No gel formation was observed and after purification, SEC analysis showed a higher molecular weight polymer had been formed, Table 1 and Fig. 3. The polymerisation of FTC with the trifunctional chloroformate 2 was conducted at a molar ratio of 1.5 : 1 respectively, to ensure a 1 : 1 ratio of reacting functional groups. This creates an average functionality value of 2.4 and an expected gel point at 83% monomer conversion. Indeed, the reaction mixture gelled and, after purification to remove all solvents, the solid product poly(FTC-TMP) was insoluble in DMF, DMSO and CHCl_3_.

Due to the low molecular weight nature of poly(hexyl-FTC), comparative analysis with poly(hexyl-FTC-TMP) was conducted using the oligomer SEC and a DMF eluent. As can be seen in Fig. 3, branched poly(hexyl-FTC-TMP) also comprised a similar distribution of low molecular weight species, but the distribution extended slightly beyond the exclusion limits of the columns that were essential to provide the resolution of the low molecular weight species. Despite this limitation, comparative *M*_n_, *M*_w_ and *Đ* values were obtained as pMMA equivalent values, Table 1. It is important to note that this analysis underestimates the actual pMMA equivalent values but serves to show the higher molecular weight of the branched polymer formed here.

**Fig. 4:**
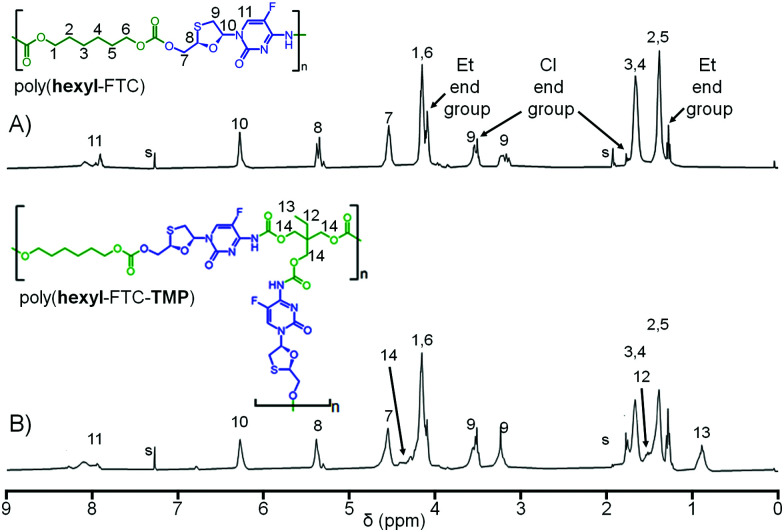
Comparative ^1^H nuclear magnetic resonance spectra (CDCl_3_) of (A) linear poly(hexyl-FTC); and (B) branched poly(hexyl-FTC-TMP). Resonances identified for ethyl carbonate end groups, intentionally created to consume unreacted chloroformate chain ends, and alkyl chloride end groups derived from degradation of chloroformate monomers.

Comparative ^1^H NMR analysis of poly(hexyl-FTC), (hexyl-FTC-TMP), and model compounds 3, 4, and 5 allowed the assignment of the key structural features of the linear and branched carbamate/carbonate polymers, Fig. 4 (Fig. S20–S23, ESI†). Importantly, the ethyl chain-ends resulting from quenching of unreacted chloroformate groups with ethanol were observed; however, additional resonances that were assigned to alkyl chloride chain-ends after comparison with literature spectra of 1,6-dichlorohexane were also seen.^28^ This is expected to be present as an impurity in the commercial sample of 1 and most likely due to intramolecular elimination on storage. ^13^C NMR analysis also showed the presence of alkyl chloride and any further development of the implants described here will need to address the presence of these potentially concerning chain-ends. No evidence of alkyl chloride functionality was seen within the model molecules 3–5, and it is only possible for these to be incorporated when using multifunctional chloroformates.

As mentioned earlier, the inspiration for the polymer synthesis strategies shown here was taken from small molecule prodrug chemistry previously reported.^16^ The model compound 5 is an example of our previous carbamate/carbonate prodrugs but FTC prodrugs with alkyl substituents ranging from 1–8 carbons were all shown to have melting points below ambient temperature and led to the formation of semi-solid prodrug nanoparticles. Differential scanning calorimetry (DSC) was used to study the solid polymers synthesised here and their glass transition temperatures (*T*_g_) were measured at the midpoint of the transition as poly(hexyl-FTC) = 28 °C, poly(hexyl-FTC-TMP) = 34 °C and poly(FTC-TMP) = 51 °C (Fig. S24, ESI†). The trend of increasing *T*_g_ from linear to branched and crosslinked architectures is consistent with an expected increasing restriction in backbone mobility. All polymers were clearly candidates for solid implant manufacture at ambient temperature.

### Confirmation of FTC release from polymer prodrugs

**Fig. 5:**
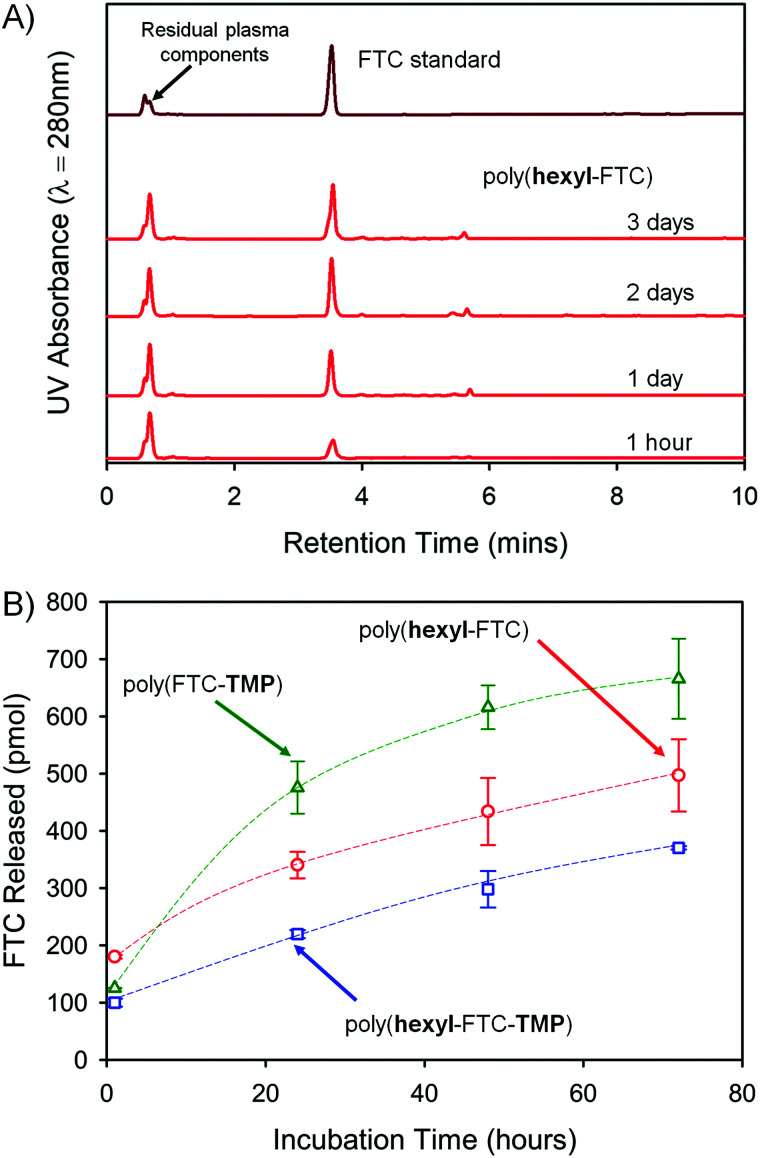
HPLC studies of FTC release from powdered FTC-containing polymers of differing architectures. (A) Demonstration of the appearance of FTC parent drug on exposure of linear poly(hexyl-FTC) to mixed gender human plasma; (B) comparative cumulative release of FTC from linear poly(hexyl-FTC) (open red circles), branched poly(hexyl-FTC-TMP) (open blue squares), and crosslinked poly(FTC-TMP) (open green triangles).

FTC release from the polymers is reliant on the cleavage of both carbamate and carbonate backbone chemical links, and fragments that may be formed include distributions of carbonate/carbamate oligomers through to dimers. The complexity of the fragmentation is difficult to fully characterise, therefore the formation of free FTC was monitored during the incubation of the three FTC-containing polymers with mixed gender human plasma at 37 °C. Each of the polymers were initially dissolved in DMSO and high-performance liquid chromatography (HPLC) was utilised to monitor FTC concentration at a UV wavelength of 280 nm over 3 days, Fig. 5A. Importantly, as a crosslinked sample, poly(FTC-TMP) was not able to be dissolved and was studied as an insoluble sample under identical conditions to those used for the DMSO-soluble linear and branched polymers. The incubation temperature was clearly below the *T*_g_ of poly(FTC-TMP) but above the *T*_g_ of both poly(hexyl-FTC) and poly(hexyl-FTC-TMP), therefore, any precipitated linear and branched polymer would be present in this study in a different physical state to the crosslinked polymer.

An FTC standard curve was created across the expected concentration range (Fig. S25, ESI†) and samples were diluted using phosphate buffered saline (PBS) with subsequent centrifugation through a 3 kDa molecular weight cut-off filter to remove proteins and any remaining solid polymer sample. The maximum FTC release in each study was set as 1 nmol and over the 3 day study period, the three polymers were observed to release up to 67% of the FTC present within the sample under these conditions (poly(hexyl-FTC) = 49.7 ± 6.3%; poly(hexyl-FTC-TMP) = 37.0 ± 0.3%; and poly(FTC-TMP) = 66.5 ± 7.0%), Fig. 5B.

The release of FTC from all polymer samples validates the polymer-prodrug strategy described here and clearly suggests that fragments of the polymer that may enter systemic circulation would be rapidly activated to release the parent NRTI drug substance. The increased release from poly(FTC-TMP) may be a function of the physical state of the sample as the erosion of a crosslinked solid during carbamate/carbonate cleavage may lead to porous structures with increasing surface area. This has not been investigated further during this study.

### Formation of implants from FTC-containing polymers

Several routes to implant manufacture exist and recent studies aiming to control the release of another NRTI, namely tenofovir alafenamide (TAF), have utilised powder filled polyurethane tubes with heat-sealed ends. The TAF implant strategy involves the use of the wall of the polyurethane tube as a semipermeable membrane to control drug release; however, local inflammation and necrosis at the implant site has been seen in primate studies^29^ and it is unclear whether these are drug, polymer of implant physical structure related.

**Fig. 6:**
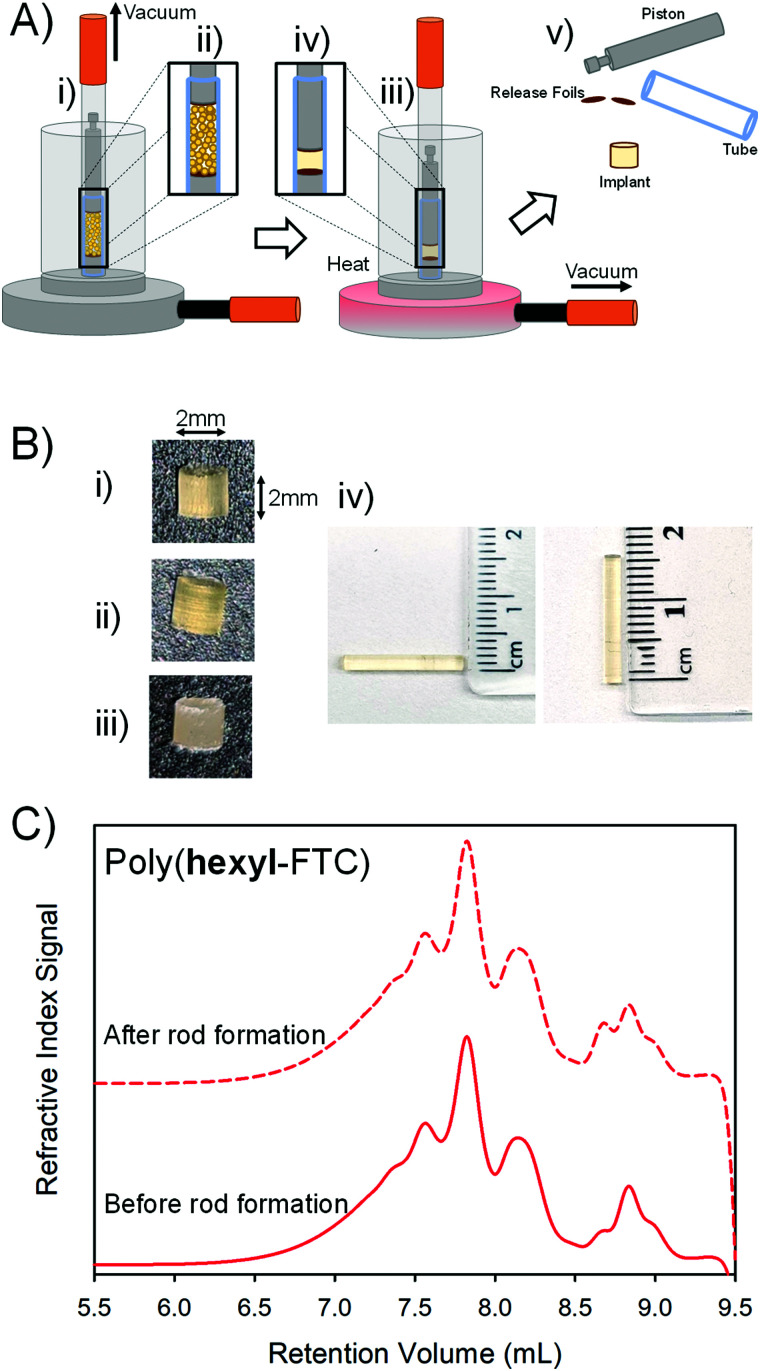
Formation of solid implants from FTC-containing polymers. (A) Schematic representation of the vacuum compression moulding process: (i) powdered polymer is added to the mould, and (ii) a vacuum is applied to simultaneously degas the powder and apply a piston to compress the sample, (iii) heating allows the powder to reach temperatures above the glass transition to form (iv) a compressed homogeneous sample which is (v) recovered after cooling as disassembly of the mould. (B) Images of 2 mm implant rods formed from (i) linear poly(hexyl-FTC), (ii) branched poly(hexyl-FTC-TMP), and (iii) crosslinked poly(hexyl-FTC-TMP) – note: crosslinked sample does not form a transparent homogeneous implant rod; (iv) images of 15 mm implant rods of poly(hexyl-FTC). (C) Comparative oligomer SEC of linear poly(hexyl-FTC) showing no apparent polymer degradation during vacuum compression moulding under these conditions.

Vacuum compression moulding (VCM) has recently been reported as a screening tool for excipients in hot-melt extrusion processes.^30^ The VCM process applies relatively low temperatures to fuse powders together into homogeneous solid structures whilst applying a vacuum to apply a 150 N force and remove gases to avoid bubble entrapment, Fig. 6.

The three FTC-containing polymers were studied under VCM conditions. In short, the powders were loaded into the VCM mould using a PTFE release foil above and below the powder, Fig. 6A(i + ii), air was removed followed by heating to 100 °C and the application of vacuum to compress the sample into a cylindrical rod, Fig. 6A(iii + iv). After cooling, the assembly was dismantled and the implant rods were removed, Fig. 6(v). Initially, 2 mm × 2 mm homogeneous rods were formed from poly(hexyl-FTC), Fig. 6B(i), and poly(hexyl-FTC-TMP), Fig. 6B(ii). To establish the scope of rod formation, poly(hexyl-FTC) and the ground crosslinked poly(FTC-TMP) were blended in a 1 : 1 ratio and processed to form translucent rods, Fig. 6B(iii), using the linear FTC-containing polymer as a novel binder for the powdered crosslinked polymer. Larger rods (15 mm × 2 mm), suitable for future *in vivo* studies, were also readily formed, Fig. 6B(iv).

The process temperature (100 °C) did not cause the linear or branched polymers to liquify and the moulding process was highly successful. To establish the heat stability of the chemistry employed here under VCM conditions, oligomer SEC was conducted on poly(hexyl-FTC) before rod formation and after VCM processing followed by grinding and dissolution. The molecular weight distributions showed no meaningful differences, Fig. 6C, and the polymers were therefore deemed to be compatible with rod formation by vacuum compression moulding.

### *In vitro* drug release from FTC-containing polymer implants

The study of FTC release in mixed gender human plasma clearly indicated the cleavage of the polymer backbone and degradation of both the carbamate and carbonate linking chemistries within the polymer prodrugs. The reduced surface area and increased bulk density of the implant rods when compared to the powdered FTC-containing polymers, and dissolved macromolecules, was considered a potential hindrance to the release of free FTC; therefore, drug release was studied in a PBS medium and compared against a PBS medium containing pooled human liver microsomes and the impact of addition of the carboxylesterase 1 (CES1) inhibitor 1,2-diphenylethane-1,2-dione (also referred to as benzil); human liver microsomes contain various carboxylesterases and cytochrome P450 metabolic enzymes, several of which are also expected to be present within target implant sites.^31,32^

**Fig. 7:**
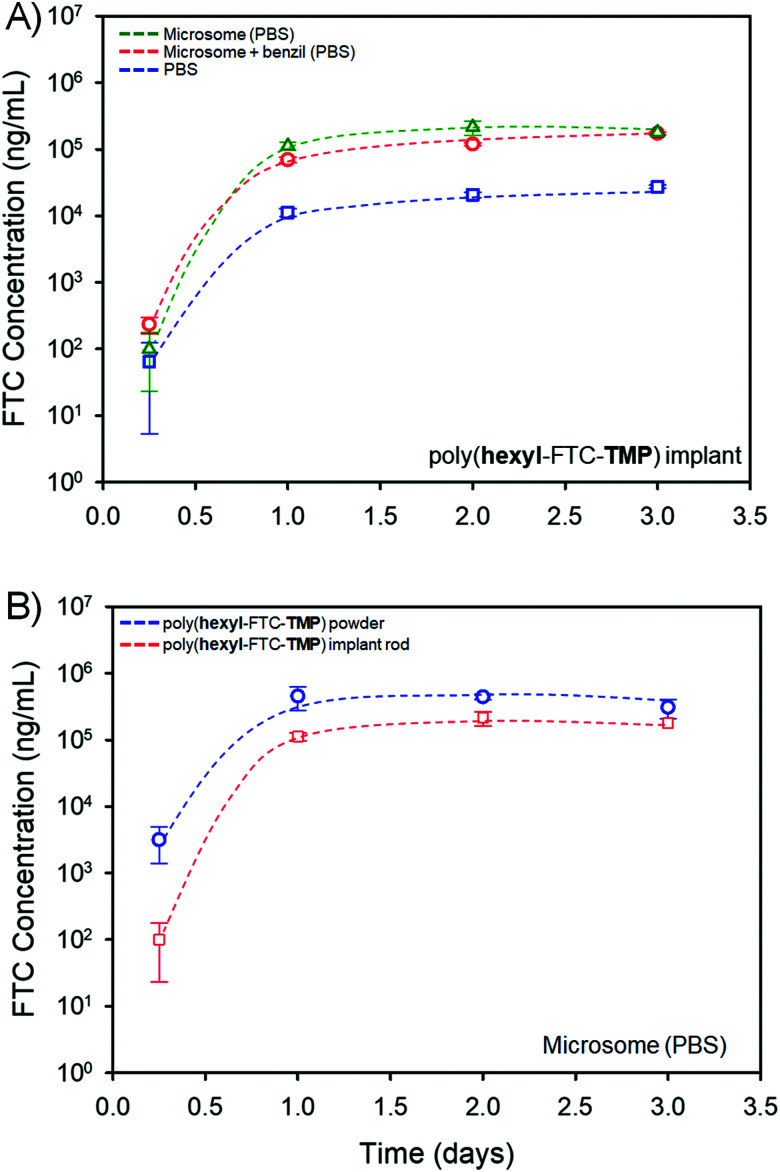
LC-MS/MS study of FTC release from a polymer implant generated from poly(hexyl-FTC-TMP). (A) Impact on cumulative FTC release in the presence of pooled human liver microsomes (open green triangles), in the presence of microsomes and the carboxylesterase inhibitor, benzil, (open red circles) and a comparative control study in PBS (open blue squares). (B) Comparative release of FTC from powdered solid polymer (open blue circles) and implants (open red squares).

Poly(hexyl-FTC-TMP) derived implant rods, Fig. 6B(ii), were selected for this study as the macromolecules contain both bifunctional and trifunctional chloroformate monomer residues and represent the chemistry of linear, branched and crosslinked polymers. The implant rods were incubated under the three conditions described above at 37 °C for 3 days with regular sampling. Analysis of the samples and creation of cumulative release curves, Fig. 7, utilised a previously validated HPLC-mass spectroscopy assay.^33^

The implant rods were shown to release FTC when incubated solely in the PBS medium, Fig. 7A, presumably through the hydrolysis of both carbonate and carbamate links under these conditions. In the presence of pooled human liver microsomes the rate of FTC noticeably increased, indicating the impact of enzymatic cleavage of the backbone. This is consistent with the studies of FTC release in mixed gender human plasma described above, and the action of liver and muscle S9 fractions on small molecule carbamate/carbonate FTC prodrugs previously reported by this team.^16^ On addition of benzil, a moderate reduction in FTC release was observed, indicating that although CES activity is contributing to the degradation of the polymer backbone, other enzymatic action may contribute to the cleavage of backbone linkers, Fig. 7A.

Comparative studies of powdered poly(hexyl-FTC-TMP) and the implant rods also showed a reduction in FTC release, as may be expected from the compacted nature of the implant and the dramatically reduced surface area available for hydrolysis and enzymatic attack. Again, the direct mechanism of, and the complex fragmentation pattern resulting from, poly(hexyl-FTC-TMP) degradation has not been determined but the data shown here underlines the potential for delayed release of FTC over several days from these polymer prodrug strategies.

## Conclusions

Water-soluble drug compounds are critical to the treatment, prophylaxis and maintenance of numerous diseases and conditions. In many cases, their water solubility has aided formulation of solid format medicines that dissolve and release the active ingredients after oral administration; however numerous drug compounds suffer from sub-optimal dosing due to issues relating to bioavailability, rapid clearance leading to short circulatory half-lives, and significant pill burden for patients. FTC is a key component of HIV therapy strategies and long-acting approaches may open opportunities for therapy optimisation.

Here the use of FTC within the synthesis of poorly water-soluble polymers containing FTC as a structural backbone component of the polymer chains was studied. The formation of varying architectures was readily achieved; however, significant impact on release of FTC cannot be directly inferred from the data presented. There are indications that branching may slow the release somewhat, but this would require significant additional study. The ready formation of solid implant rods demonstrated the value of vacuum compression moulding in early-stage development of implantable structures using the study polymers. Importantly, implant formation did not prevent FTC release and sustained release was seen over a 3 day period in these *in vitro* studies. Longer studies *in vitro* and *in vivo* will be required to determine the ultimate utility of this approach but the presented data clearly highlight the potential of this chemical strategy. Drug-eluting implants are known to induce granuloma formation^34^ as a foreign body response, and these have also been observed when using nondegradable polymer microspheres^35^ and metal implants.^36^ Granuloma formation has been shown to impact drug release from bioerodible implants,^37^ further extending the duration of circulating therapeutic drug concentrations. It is unknown whether these FTC-containing implants would also induce granuloma formation or whether this would aid the attainment of relevant *in vivo* release kinetics.

Many drug-eluting implants require removal and replacement, but the FTC-containing implant candidates presented here are expected to fully degrade and avoid the need for a surgical removal process. The implants are highly drug loaded (>57 wt%) due to the use of FTC as a monomer and optimisation towards clinically-relevant drug exposure durations is a clear next step after proof-of-concept of *in vivo* studies of extended drug release. Two additional important factors that require consideration are: (1) optimisation of the implant physical properties may be required as currently the rods are robust but undergo brittle fracture and larger implants will be needed for future human evaluation; and (2) although the implants will not experience varying pH after administration, the impact of pH on the carbamate and carbonate linking groups^39,40^ will require study to avoid manufacturing issues. The macromolecular approach presented here may be applicable to numerous functional drug compounds including the use of two or more drug substances in statistical copolymers or the blending of different homopolymers within combination implants.

## Experimental

### Materials

Anhydrous 1,6-hexamethylene bis(chloroformate) (95%) and trimethylolpropane tri(chloroformate) (97%) were purchased from Manchester Organics Ltd (UK) and used as received. 4-Dimethylaminopyridine (DMAP), anhydrous pyridine, anhydrous dimethylsulfoxide (DMSO) and anhydrous dichloromethane (DCM) were purchased from Merck (UK) and used as received. *N*,*N*′-Dimethylformamide (DMF), ethanol, ethyl acetate, hexane, CDCl_3_, and magnesium sulfate (MgSO_4_) were purchased from Fischer Scientific, whilst anhydrous ethanol and hexyl chloroformate was purchased from Sigma Aldrich. Emtricitabine (FTC) was purchased from WISCHEM (China) and used without further purification. Male and female human plasma and the dipotassium salt of ethylenediaminetetraacetic acid (K2-EDTA) were purchased from BioIVT and the plasma samples were pooled. Benzil was purchased from Merck (Germany). Pooled, mixed gender, adult human liver microsome and phosphate buffered saline (PBS) at pH 7.4 was purchased from ThermoFisher Scientific (USA). Carboxylesterase 1 (CES1) specific activity assay kit was purchased from Abcam (UK).

### Methods

#### Characterisation

Nuclear magnetic resonance spectroscopy (NMR): ^1^H and ^13^C NMR was conducted at 400 MHz and 100 MHz respectively, using a Brucker Avance III HD NMR spectrometer. Samples were acquired in CDCl_3_ and chemical shifts were recorded in parts per million (ppm) with reference to the solvent peak of CDCl_3_ (^1^H = 7.26 ppm, ^13^C = 77.16 ppm). Electrospray mass spectrometry (EI-MS) analysis: All studies were conducted using an Agilent 7200 mass spectrometer with samples prepared in acetonitrile. Fourier transform infra-red spectroscopy: studies were carried out using a Vertex 70 FT-IR spectrometer equipped with germanium crystal and in attenuated total reflection mode at ambient temperature. Size exclusion chromatography (SEC): single detection SEC was conducted using an Agilent 1260 Infinity II equipped with a PLgel Mixed-E column (300 mm × 7.5 mm, molecular weight range = 1000–30 000 g mol^−1^), a guard column and an RI detector. A DMF mobile phase containing LiBr (0.01 M) at 60 °C was used with a flowrate of 1 mL min^−1^. Number average (*M*_n_) and weight average (*M*_w_) molecular weights and dispersity (*Đ*) were calculated from a standard calibration curve using poly(methyl methacrylate) (pMMA) standards with *M*_p_ values ranging from 850–27 600 g mol^−1^. Differential scanning calorimetry (DSC): thermal analysis of polymer samples was obtained using a TA Instruments Discovery DSC calibrated using an indium standard and an aluminium reference pan. Samples were prepared in Tzero® pans using a Tzero® press and measured under a nitrogen atmosphere at 0.1 MPa. Polymers were heated to 100 °C at a rate of 5 °C min^−1^ to erase thermal history, before cooling to -50 °C at a rate of 10 °C min^−1^. The polymers were then heated to 100 °C at a rate of 5 °C min^−1^ to determine glass transition temperature, *T*_g_, values. Sample purification: small molecule model compounds were isolated using a Buchi Pure C-815 Flash Chromatography system equipped with a FlashPure prepacked silica cartridge (12 g) and a mobile phase consisted of ethyl acetate/hexane using a polarity gradient (30 minutes) increasing from 10% v/v ethyl acetate to 100% ethyl acetate. Implant preparation: Implant rods were prepared using a Melt Prep vacuum compression moulding tool consisting of heating and cooling units and a vacuum pump. A low-pressure lid was used to prepare the 2 mm × 2 mm rods using a 2 mm diameter polytetrafluoroethylene (PTFE) tube and 2 mm PTFE release foils. FTC release studies in mixed gender human serum: Samples were analyzed using an Agilent 1260 Infinity II HPLC. Solvent A: 50 mM ammonium acetate pH 6.0, solvent B: HPLC grade acetonitrile. Method: 5% B for 1 min, 5–100% B over 15 min, 1 min 100% B. FTC was detected at 280 ± 2 nm. Peak areas were integrated and converted to picomoles using the standard curve of FTC, which was generated by serial dilution of FTC in DMSO and measurement of peak areas corresponding to FTC. Peak area *versus* picomoles of FTC was plotted. The standard curve was conducted in duplicate. FTC release studies in the presence of microsomes: detection of FTC was performed using a previously validated liquid chromatography mass spectrometry (LC-MS/MS) method^33^ employing a SCIEX 6500+ QTRAP which operated in negative mode. Separation of samples was conducted using a Kinetex® F5 column (2.1 × 100 mm, 2.6 μm). The mobile phases consisted of water with acetic acid (0.1%) and methanol with acetic acid (0.1%).

### Synthesis

#### Synthesis of model compounds 3, 4 and 5

FTC (2.013 g, 8.14 mmol), anhydrous pyridine (0.72 mL, 9.10 mmol) and anhydrous DCM (35 mL) were added to a 100 mL round-bottom flask, fitted with stirrer bar and nitrogen inlet. The reaction flask was placed in an ice bath and hexyl chloroformate (1.47 g, 8.93 mmol) diluted in DCM (5 mL) was added by slow, dropwise addition. After complete addition, the reaction mixture formed a yellow precipitate. The reaction was left to stir and warm to room temperature overnight. After 18 hours, the yellow precipitate had dissolved, forming a clear yellow solution. The reaction medium was concentrated on a rotary evaporator to an approximate volume of 20 mL and the crude mixture was purified by flash chromatography. Model compound 3 was isolated when the ethyl acetate/hexane solvent gradient consisted of 27% ethyl acetate, and 5 was isolated at 54% ethyl acetate. The solvents were removed *in vacuo* and both products appeared to be a yellow semi-solid nature. Yield = 20% and 13.5% for 3 and 5 respectively.

4 was synthesised using a modification to the above method as follows: FTC (1 eq.), DMAP (1 eq.) and pyridine (30 eq.) were added to DCM (50 mL) under nitrogen and chilled in an ice water bath for 10 min. Hexyl chloroformate was added to the stirring solution (1 eq.). The reaction was monitored by TLC and was deemed complete after 16 hours with the disappearance of spots relating to FTC. The crude product was diluted with DCM (200 mL) and washed with HCl (1 M, 2 × 100 mL), followed by brine (2 × 100 mL) and dried over anhydrous MgSO_4_. The recovered materials were further purified by automated flash chromatography (solvent 30–70% EtOAc in n-hexane over 30 min).

3: ^1^H NMR (400 MHz, chloroform-*d*) *δ* (ppm) 8.56 (dd, *J* = 39.6, 6.4 Hz, 1H), 6.22 (d, *J* = 4.2 Hz, 1H), 5.31–5.23 (m, 1H), 4.28–4.08 (m, 3H), 3.96 (dd, *J* = 12.8, 3.1 Hz, 1H), 3.50 (dd, *J* = 12.5, 5.3 Hz, 1H), 3.22 (dd, *J* = 12.6, 3.1 Hz, 1H), 1.75–1.56 (m, 2H), 1.29 (dqd, *J* = 14.8, 7.6, 4.7, 3.8 Hz, 6H), 0.93–0.76 (m, 3H). ^13^C NMR (101 MHz, chloroform-*d*) *δ* (ppm) 153.71, 153.54, 88.72, 87.41, 66.80, 63.09, 62.24, 38.91, 32.76, 31.69, 31.48, 28.62, 28.59, 25.52, 22.68, 22.59, 14.07. (Found (%) C, 47.09; H, 5.88; N, 11.26. Calculated (%) C, 47.99; H, 5.91; N, 11.19).

4: ^1^H NMR (500 MHz, chloroform-*d*) *δ* (ppm) 8.67 (s, 1H), 7.83 (d, *J* = 6.4 Hz, 1H), 6.29 (ddd, *J* = 5.6, 4.0, 1.7 Hz, 1H), 5.84 (s, 1H), 5.35 (dd, *J* = 4.4, 3.0 Hz, 1H), 4.52 (qd, *J* = 12.3, 3.7 Hz, 2H), 4.16 (td, *J* = 6.7, 1.8 Hz, 2H), 3.52 (dd, *J* = 12.2, 5.3 Hz, 1H), 3.11 (dd, *J* = 12.2, 4.0 Hz, 1H), 1.70–1.62 (m, 2H), 1.40–1.22 (m, 6H), 0.91–0.81 (m, 3H). ^13^C NMR (126 MHz, chloroform-*d*) *δ* (ppm) 158.53, 158.41, 154.99, 153.72, 137.52, 135.59, 125.41, 125.15, 87.59, 83.57, 66.87, 38.46, 31.42, 28.59, 25.33, 22.56, 14.05.

5: ^1^H NMR (400 MHz, chloroform-*d*) *δ* (ppm) 8.20–7.84 (m, 1H), 6.27 (s, 1H), 5.37 (t, *J* = 3.4 Hz, 1H), 4.53 (d, *J* = 3.3 Hz, 2H), 4.15 (t, *J* = 6.8 Hz, 4H), 3.53 (dd, *J* = 12.3, 5.6 Hz, 1H), 3.17 (d, *J* = 3.8 Hz, 1H), 1.66 (q, *J* = 7.3 Hz, 5H), 1.30 (dqt, *J* = 18.5, 7.3, 4.8, 3.4 Hz, 14H), 0.86 (q, *J* = 4.7 Hz, 7H). ^13^C NMR (101 MHz, chloroform-*d*) *δ* (ppm) 154.91, 153.52, 153.34, 87.54, 84.30, 69.10, 38.73, 31.50, 31.38, 28.62, 28.52, 25.55, 25.29, 22.59, 22.55, 14.07, 14.04. (Found (%) C, 51.69; H, 6.79; N, 8.40. Calculated (%) C, 52.47; H, 6.81; N, 8.34.)

### General step-growth polymerisation method using FTC and multifunctional chloroformate monomers

FTC (1 eq.), DMAP (0.5 eq.) and pyridine (2.2 eq.) was added to a round-bottom flask fitted with a stirrer bar. DCM (approx. 1 mL) was added, the flask was placed under a nitrogen atmosphere and cooled in an ice bath. The multifunctional chloroformate monomer (1 eq.) was diluted in DCM (2 mL) and added dropwise over 25 minutes. The reaction mixture became progressively more viscous, and a yellow colouration appeared. The reaction was left to approach ambient temperature overnight (18 hours) after which time anhydrous ethanol (2 mL) was added dropwise to the mixture, followed by stirring for a further 2 hours. The reaction turned cloudy and white upon addition of ethanol; however, the reaction mixture became homogenous on stirring.

The polymer was purified by dilution of the crude reaction mixture in DCM followed by liquid–liquid extraction using HCl (1 M) and then brine. The product was isolated in the organic phase which was subsequently dried over anhydrous MgSO_4_. After removal of MgSO_4_, the solvent was removed *in vacuo*. Each polymer was synthesised using this general method but with the following specific conditions:

Poly(hexyl-FTC)–FTC (1.5 g, 6.07 mmol), DMAP (0.37 g, 3.03 mmol), anhydrous pyridine (1.08 mL, 13.3 mmol), 1,6-hexamethylene bis(chloroformate) (1.475 g, 6.06 mmol) and ethanol (2 mL). ^1^H NMR (400 MHz, chloroform-*d*) *δ* (ppm) 8.23–7.75 (m, 7H), 6.27 (d, *J* = 4.6 Hz, 6H), 5.46–5.32 (m, 5H), 5.29 (s, 1H), 4.53 (dd, *J* = 7.7, 4.5 Hz, 12H), 4.31–3.99 (m, 31H), 3.95 (dd, *J* = 12.7, 3.3 Hz, 1H), 3.88–3.80 (m, 1H), 3.51 (dt, *J* = 12.2, 6.1 Hz, 9H), 3.18 (ddt, *J* = 25.0, 12.4, 4.1 Hz, 8H), 1.92 (s, 2H), 1.65 (q, *J* = 6.9 Hz, 32H), 1.53–1.13 (m, 35H). ^13^C NMR (101 MHz, chloroform-*d*) *δ* (ppm) 157.93, 155.39, 154.91, 154.85, 153.49, 137.67, 135.25, 125.77, 125.45, 87.48, 83.94, 68.67, 67.83, 66.41, 63.90, 44.98, 38.62, 32.40, 30.75, 28.52, 28.41, 25.41, 25.36, 25.31, 14.32.

Poly(hexyl-FTC-TMP)–FTC (1.5 g, 6.07 mmol), DMAP (0.37 g, 3.03 mmol), anhydrous pyridine (1.08 mL, 13.3 mmol), trimethylolpropane tri(chloroformate) (0.488 g, 1.52 mmol), 1,6-hexamethylene bis(chloroformate) (1.11 g, 4.55 mmol) and ethanol (3 mL). ^1^H NMR (400 MHz, chloroform-*d*) *δ* (ppm) 8.07 (s, 1H), 6.35–6.14 (m, 1H), 5.46–5.25 (m, 1H), 4.69–4.45 (m, 2H), 4.13 (pd, *J* = 13.5, 8.8, 6.1 Hz, 5H), 3.52 (dt, *J* = 13.3, 4.7 Hz, 2H), 3.23 (q, *J* = 12.8, 12.3 Hz, 1H), 1.95–1.58 (m, 4H), 1.55–1.31 (m, 4H), 1.27 (t, *J* = 7.2 Hz, 1H), 1.04–0.77 (m, 1H). ^13^C NMR (101 MHz, chloroform-*d*) *δ* (ppm) 154.81, 139.45, 106.65, 87.41, 84.49, 72.12, 68.66, 67.77, 66.36, 63.86, 44.98, 44.91, 40.16, 38.56, 32.40, 32.35, 28.53, 28.42, 28.36, 26.47, 26.36, 26.12, 25.36, 25.26, 25.19, 25.14, 25.11, 24.90, 14.27, 14.22, 7.28.

Poly(FTC-TMP)–FTC (1.5 g, 6.07 mmol), DMAP (0.37 g, 3.03 mmol), anhydrous pyridine (1.08 mL, 13.3 mmol), trimethylolpropane tri(chloroformate) (1.3 g, 4.05 mmol) and ethanol (3 mL). The viscous gelled product was rinsed with HCl (aq) (3 × 100 mL) and brine (3 × 100 mL) and freeze-dried overnight.

### FTC release from polymers – study methods

#### HPLC based assay in mixed gender hPlasma

Polymers were dissolved in DMSO to obtain stock solutions containing an equivalent FTC concentration of 20 mM, calculated based on w/w% FTC in the polymer solid. Each polymer (5 μL of stock) was added to hPlasma (M + F, 95 μL) to give FTC at a final concentration of 1 mM, and the mixture was incubated at 37 °C. Aliquots (10 μL, equivalent to 10 nmol FTC) were withdrawn from the mixture at 1 h, 24 h, 48 h and 72 h, and added to 190 μL of 100 mM PBS pH 7.4. The diluted mixture was centrifuged at 14 000 rpm through 3 kDa MWCO centrifugal filters for 10 min to separate soluble FTC-containing fragments from proteins. The filtrate (20 μL) was injected then analysed using HPLC.

#### LC-MS/MS based assay using human liver microsomes

Powdered or implant rod samples of poly(hexyl-FTC-TMP), with an average mass of approximately 8 mg (equivalent to approximately 4.9 mg of FTC), were incubated at 37 °C, 250 rpm for 3 days with either 1 mL of PBS in isolation, 1 mL of 1 mM benzil with 125 μg mL^−1^ of microsome in PBS, or 125 μg mL^−1^ of microsome in PBS alone. The carboxylesterase CES1 concentration in the 125 μg mL^−1^ microsome solution was determined to be 8.5 μg mL^−1^, quantified using a CES1 specific activity assay kit, following the manufactures protocol.^38^ 500 μL samples were taken at 4 hours, 1 day, 2 days and 3 days. In order to maintain sink conditions, after each sampling time point 500 μL of fresh PBS, benzil and microsome or microsome and PBS was added to each sample as appropriate. The FTC concentration within all samples was quantified using an adapted, previously validated liquid chromatography mass spectrometry (LC-MS/MS) method.^33^

## Author contributions

S. P. R. conceptualised the polymer chemistry, supervised the experimental polymer and materials chemistry, wrote the draft and edited the final manuscript. S. P. R., A. O., C. F. M. wrote the grant proposal and secured the funding. A. S., F. Y. H., C. L. carried out the synthetic work and characterisation. P. C. and T. O. M. co-supervised the synthetic programme with S. P. R. C. F. M. supervised the analysis of the materials by HPLC methodology in the presence of mixed human plasma; K. T. conducted these experiments. A. O. and N. L. supervised the analysis of the materials by LC-MS/MS methodologies in the presence of microsomes; M. N. conducted these experiments and analysed the data.

## Conflicts of interest

S. P. R., A. O., F. H., C. L., A. S., M. N., C. F. M. and K. T. are coinventors on a published patent application (WO2020128525) describing the presented polymers.

## Supplementary Material

TB-010-D2TB00825D-s001
