# Supplementary material for: Linear and branched polymer prodrugs of the water-soluble nucleoside reverse-transcriptase inhibitor emtricitabine as structural materials for long-acting implants
Source: J Mater Chem B. 2022 May 19;10(23):4395–404. doi: 10.1039/d2tb00825d (PMC9199480; doi:10.1039/d2tb00825d)
Supplement: TB-010-D2TB00825D-s001 [file TB-010-D2TB00825D-s001.pdf]

Electronic Supplementary Material (ESI) for Journal of Materials Chemistry B.
This journal is © The Royal Society of Chemistry 2022

Linear and branched polymer prodrugs of the water-soluble nucleoside reverse-transcriptase inhibitor emtricitabine as structural materials for long-acting implants

Anika Shakil, Faye Y. Hern, Chung Liu, Kartik Temburnikar, Pierre Chambon, Neill Liptrott, Tom O. McDonald, Megan Neary, Andrew Owen, Caren Freel Meyers and Steve P. Rannard.*

*Corresponding author: E-mail: srannard@liv.ac.uk

Supplementary Information

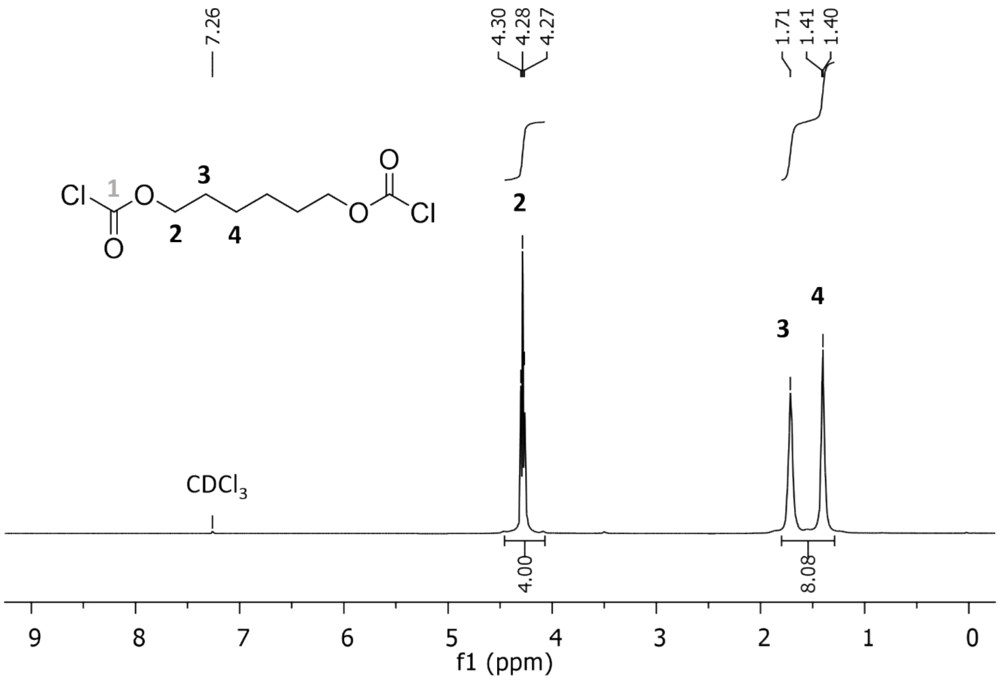

Figure S1. ^{1}H NMR ($CDCl_3$, 400 MHz) of hexamethylene bischloroformate.

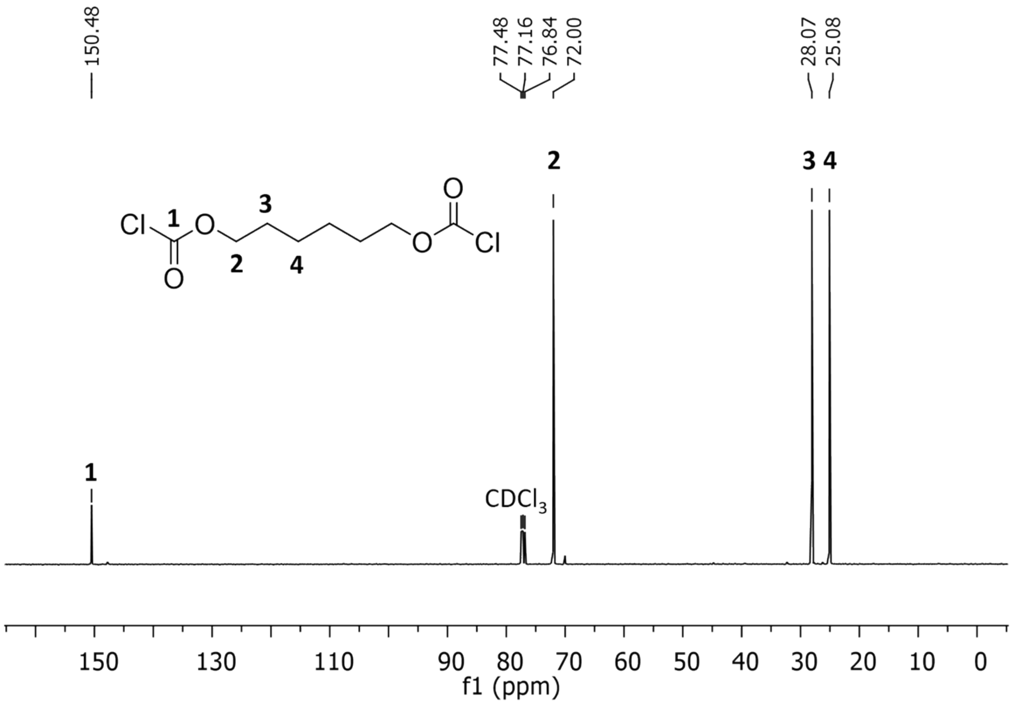

Figure S2. ^{13}C NMR ($CDCl_3$, 100 MHz) of hexamethylene bis(chloroformate).

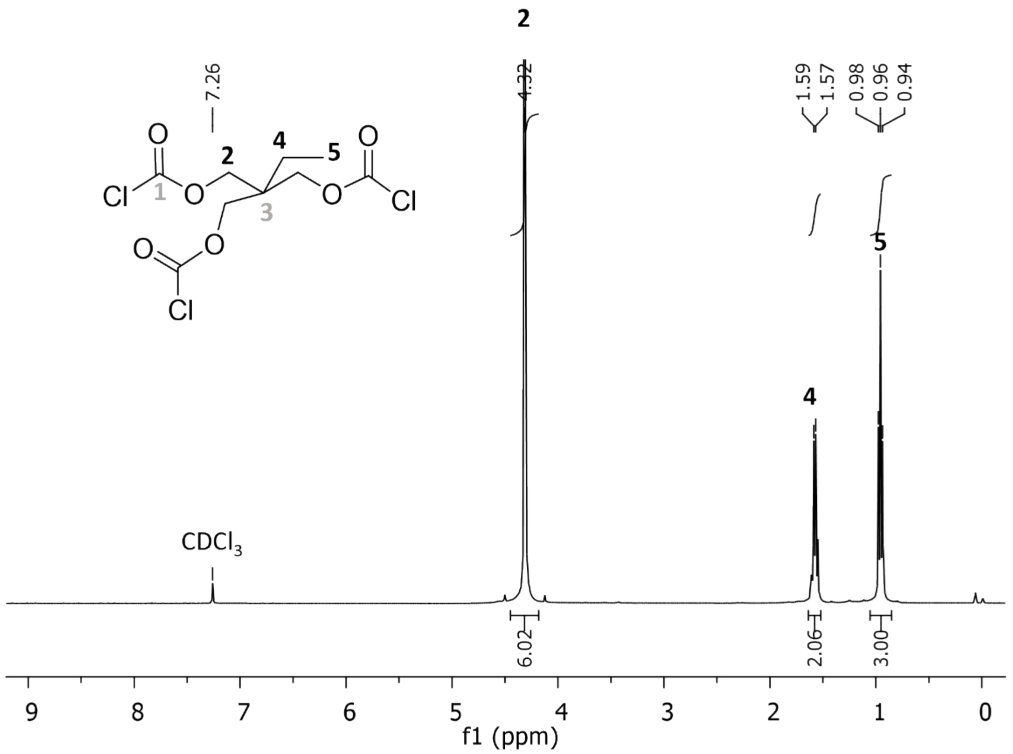

Figure S3. ^{1}H NMR ($CDCl_3$, 400 MHz) of trimethylol propane tris(chloroformate).

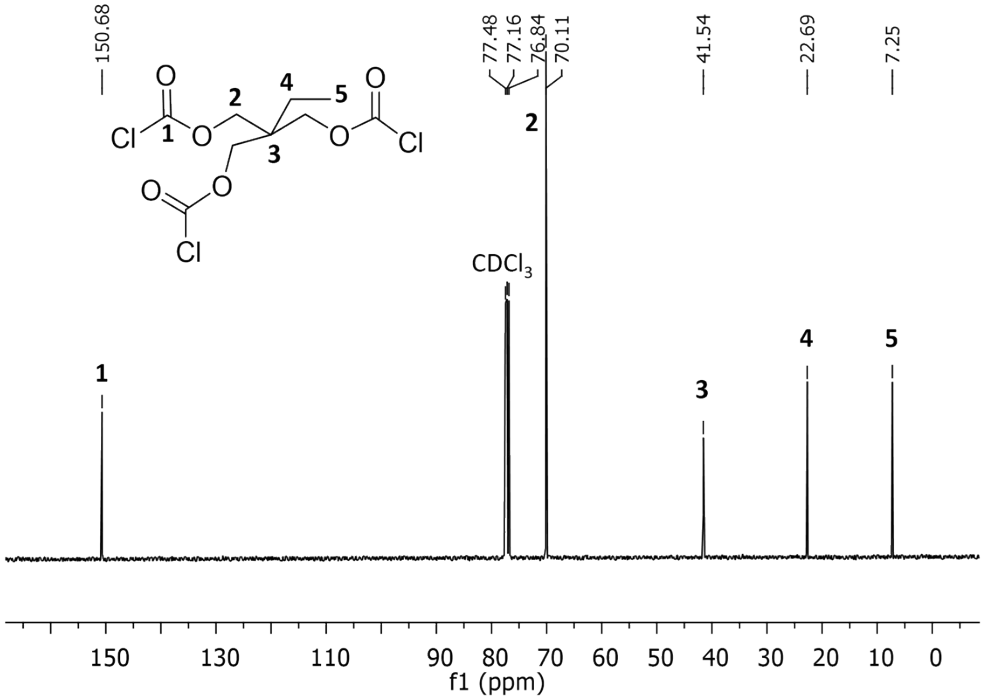

Figure S4. ^{13}C NMR ($CDCl_3$, 100 MHz) of trimethylol propane tris(chloroformate).

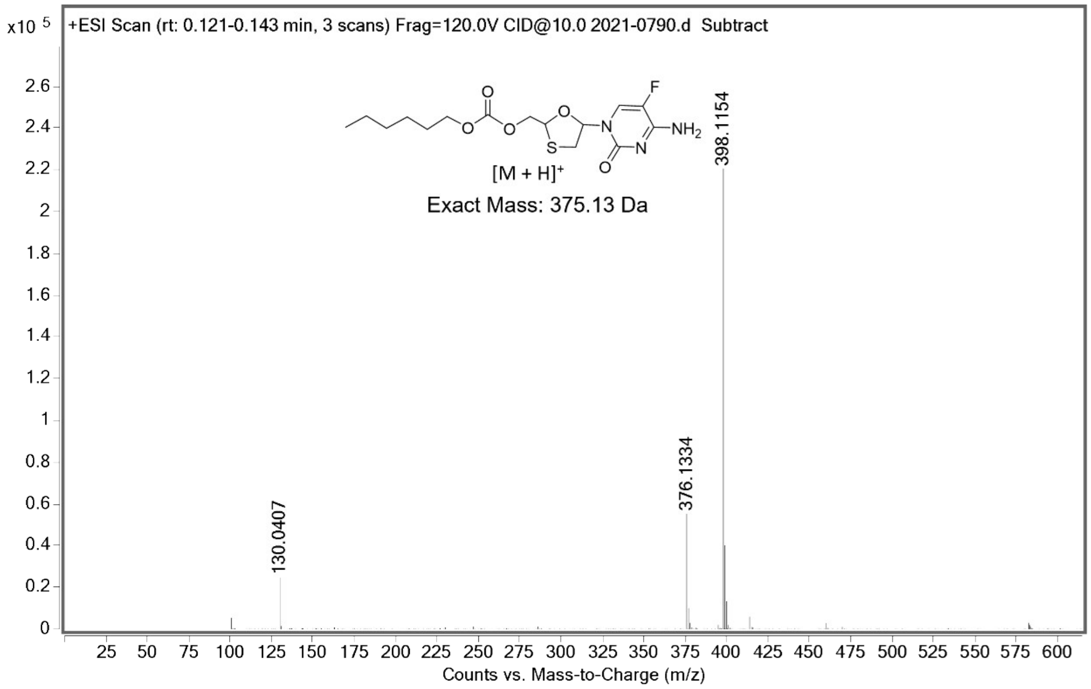

*Figure S5. ESI spectrum of the monosubstituted carbonate FTC model compound **4** (positive ion mode).*

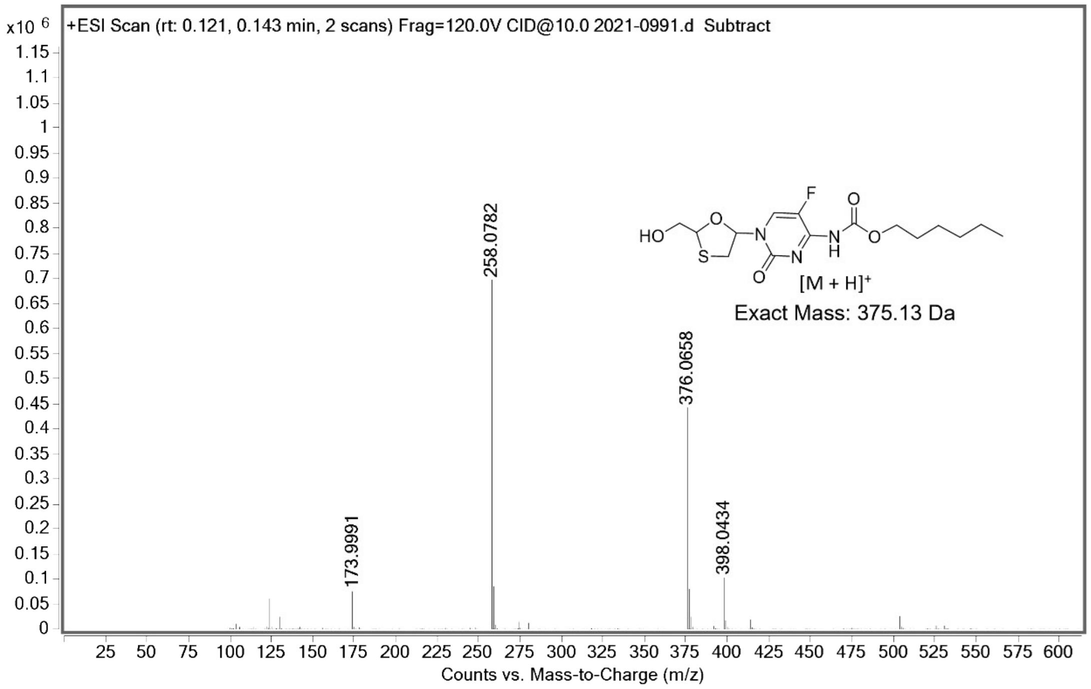

*Figure S6. ESI spectrum of the monosubstituted carbamate FTC model compound **3** (positive ion mode).*

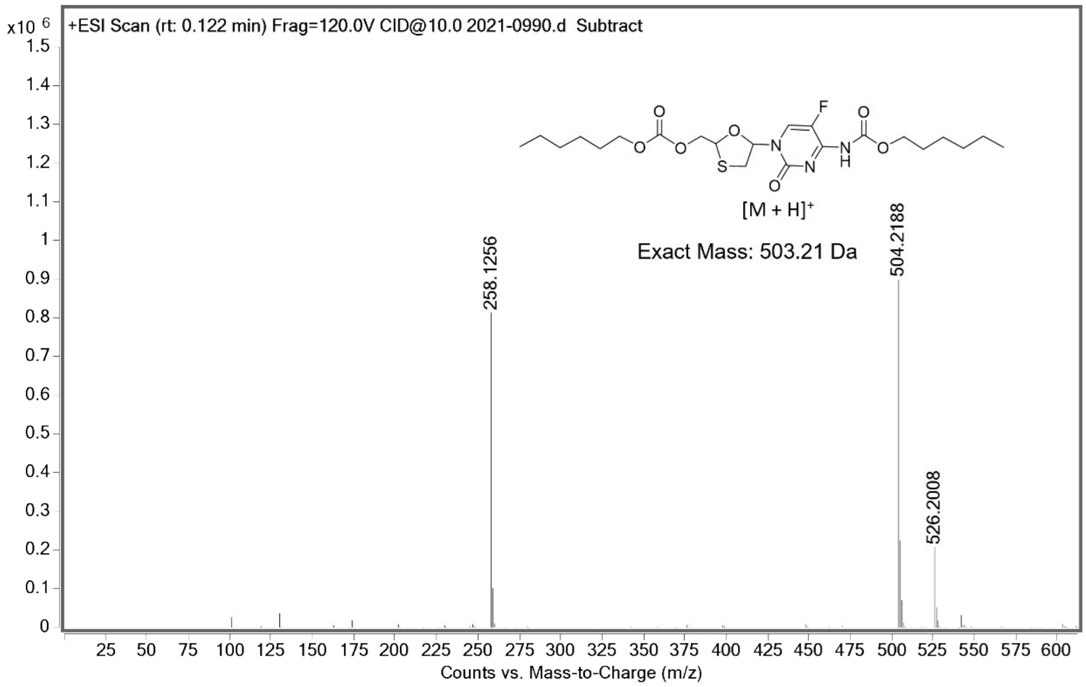

*Figure S7. ESI spectrum of the diisubstituted carbonate/carbamate FTC model compound **5** (positive ion mode)..*

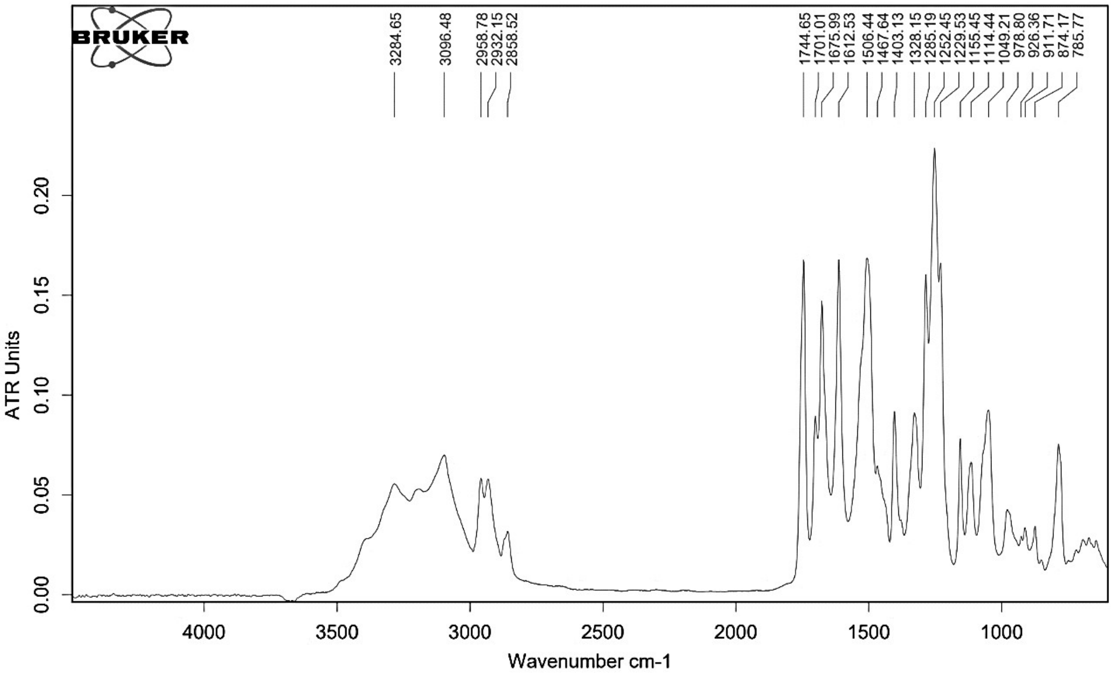

*Figure S8. FTIR spectrum of monosubstituted carbonate FTC model compound **4**.*

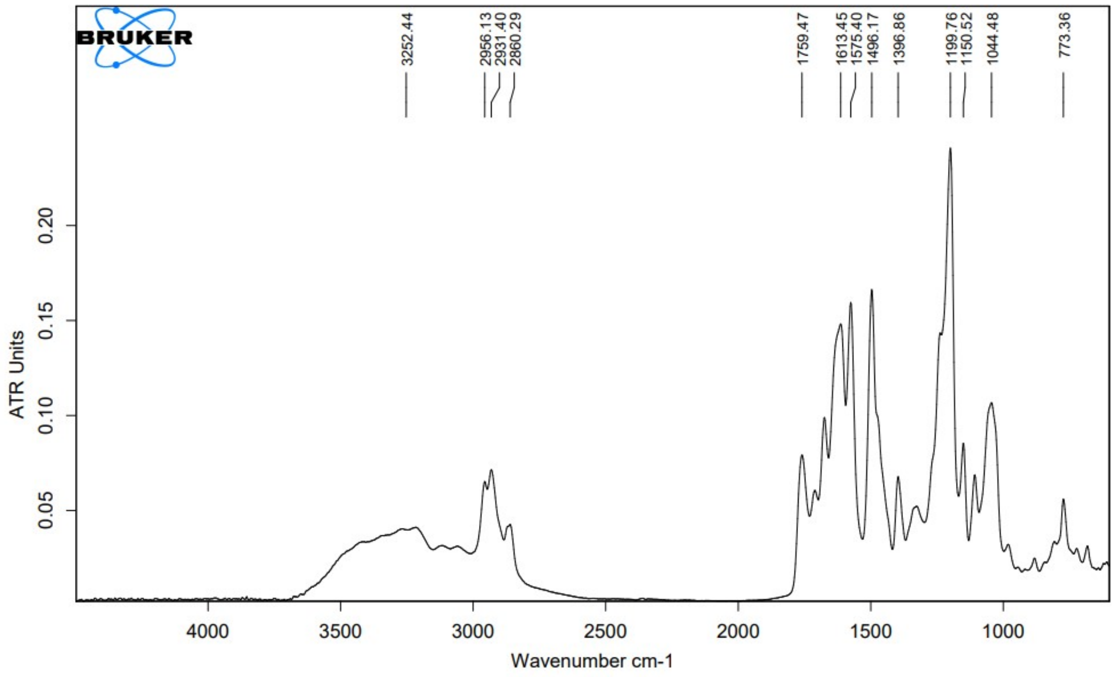

*Figure S9. FTIR spectrum of monosubstituted carbamate FTC model compound **3**.*

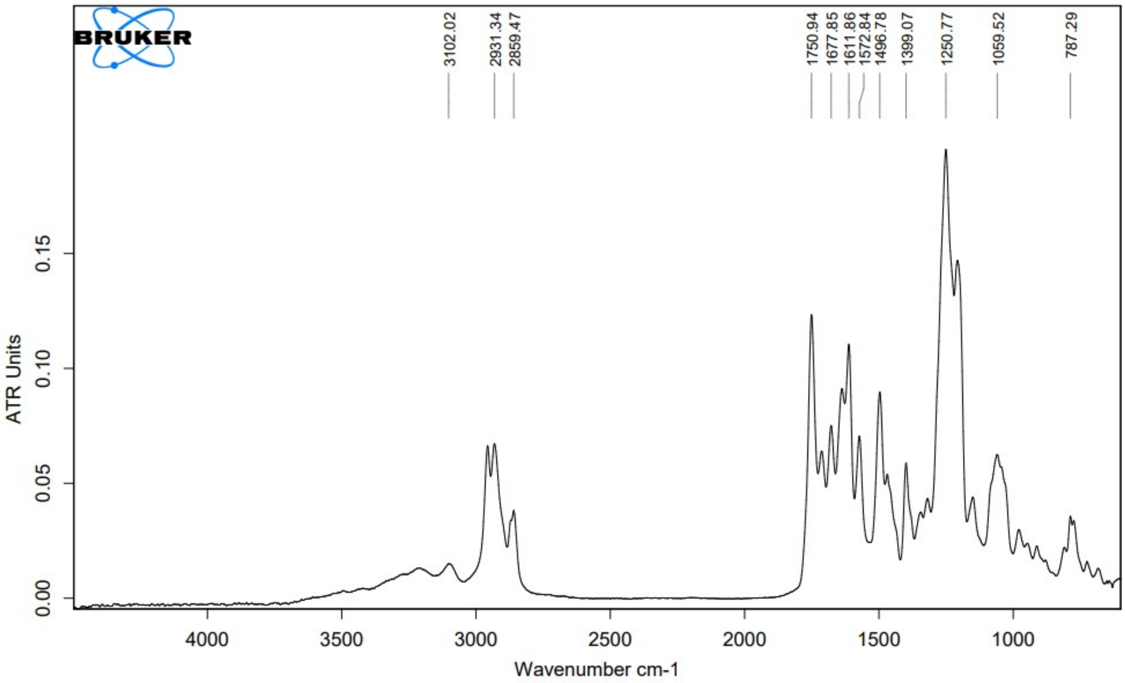

*Figure S10. FTIR spectrum of disubstituted carbamate/carbonate FTC model compound **5**.*

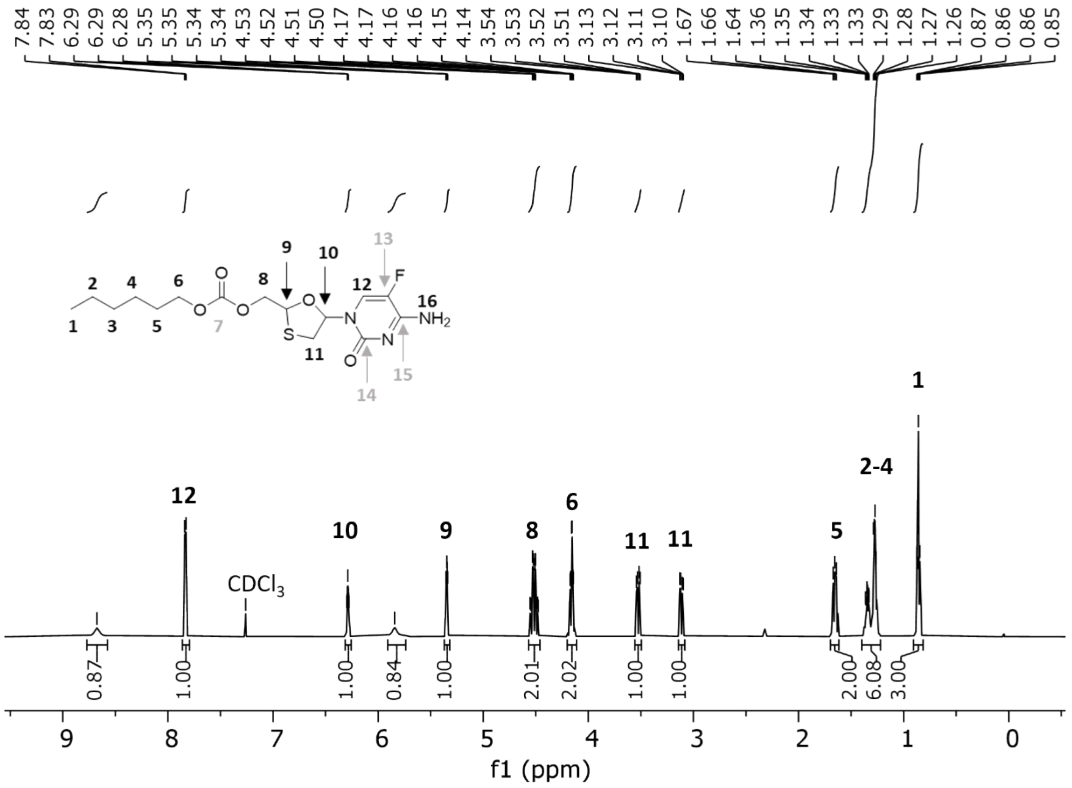

*Figure S11. ^{1}H NMR ($CDCl_3$, 400 MHz) of monosubstituted carbonate FTC model compound **4**.*

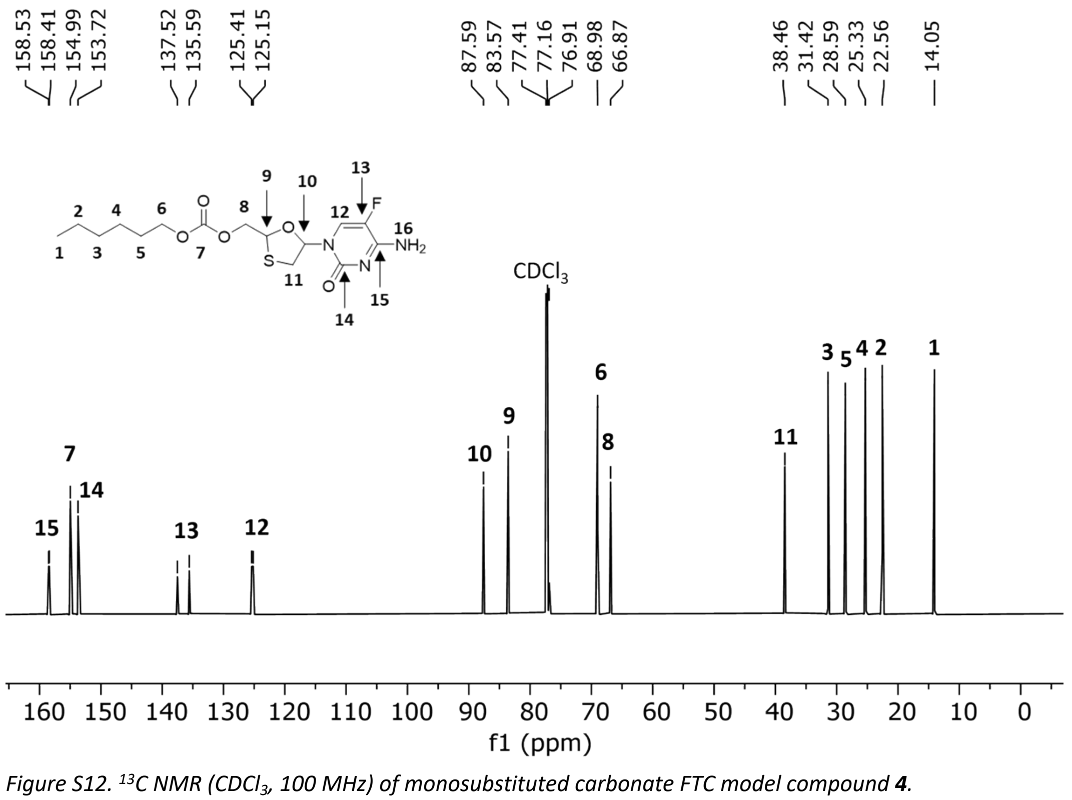

*Figure S12. ^{13}C NMR ($CDCl_3$, 100 MHz) of monosubstituted carbonate FTC model compound **4**.*

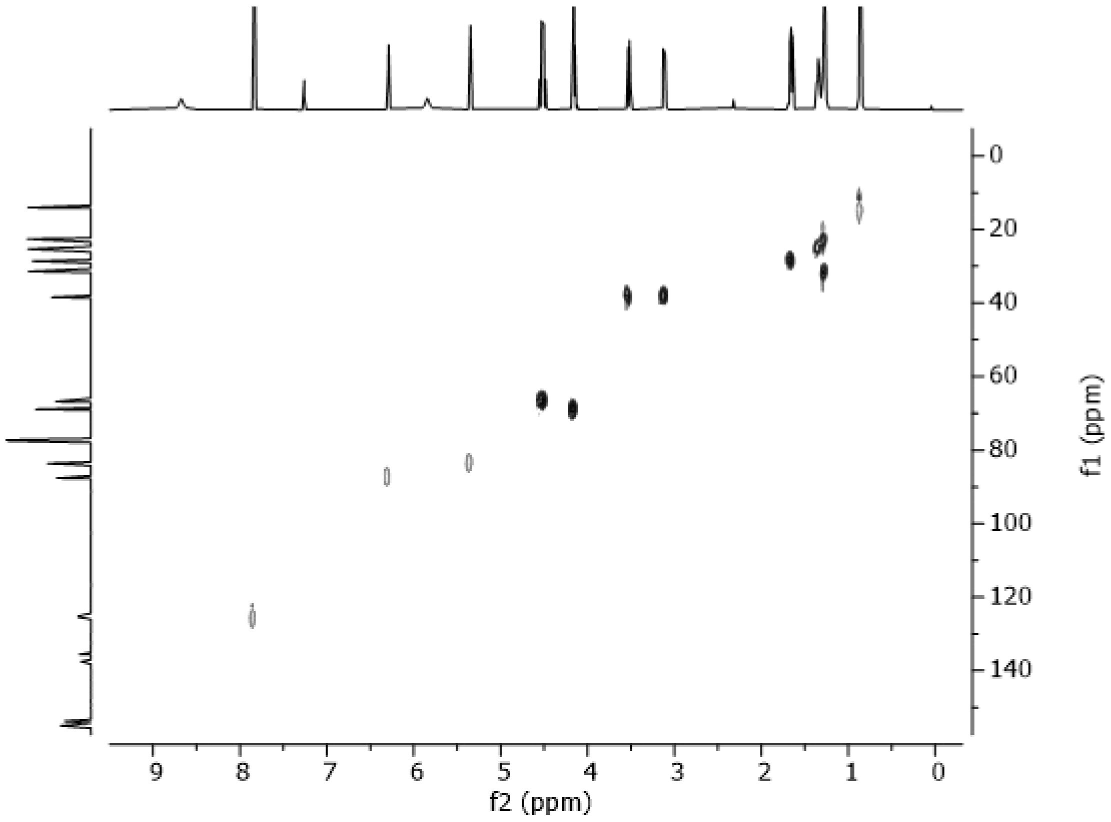

*Figure S13. HSQC ($CDCl_3$, 400 MHz) of monosubstituted carbonate FTC model compound **4**.*

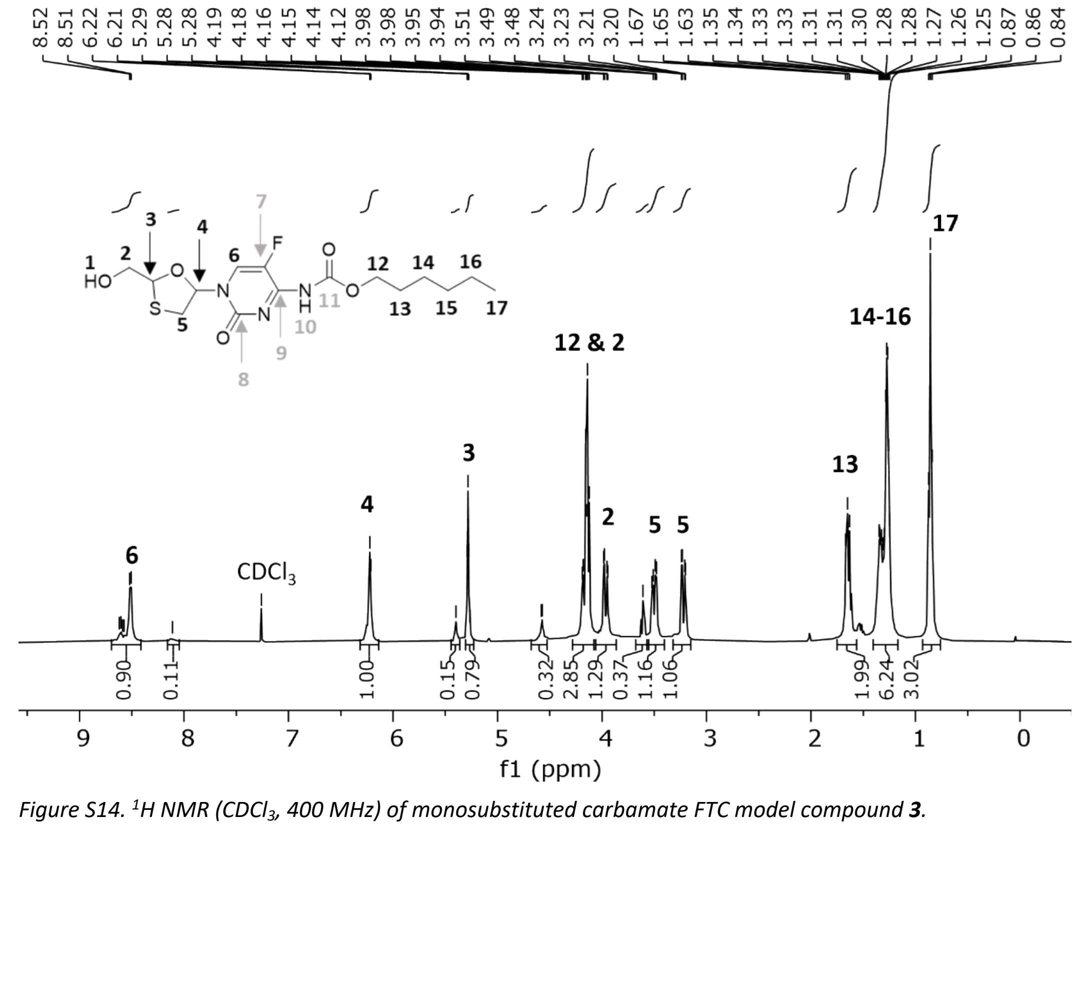

*Figure S14. ^1H NMR ($CDCl_3$, 400 MHz) of monosubstituted carbamate FTC model compound **3**.*

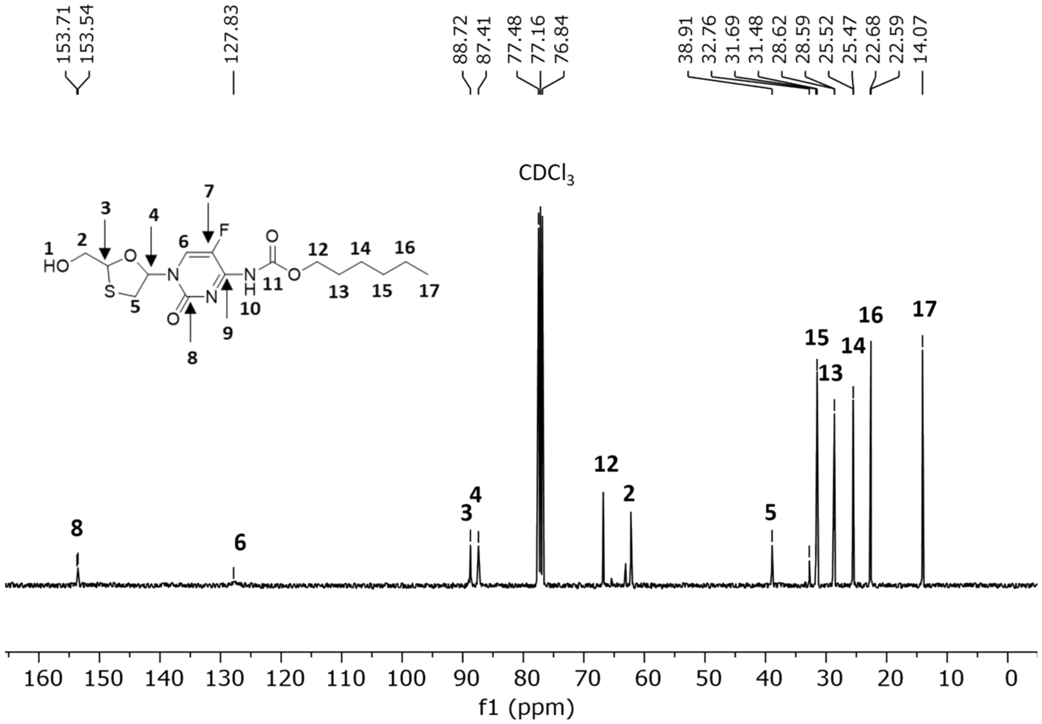

*Figure S15. ^{13}C NMR ($CDCl_3$, 100 MHz) of monosubstituted carbamate FTC model compound **3**.*

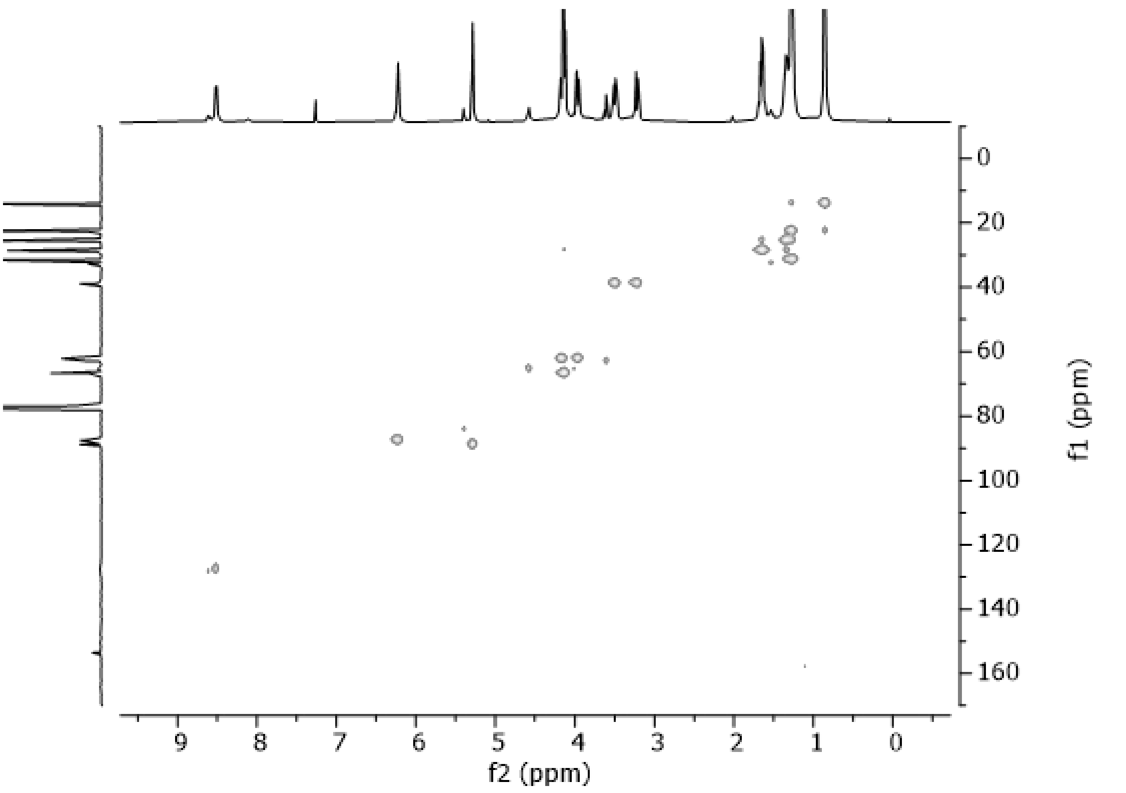

*Figure S16. HSQC ($CDCl_3$, 400 MHz) of monosubstituted carbamate FTC model compound **3**.*

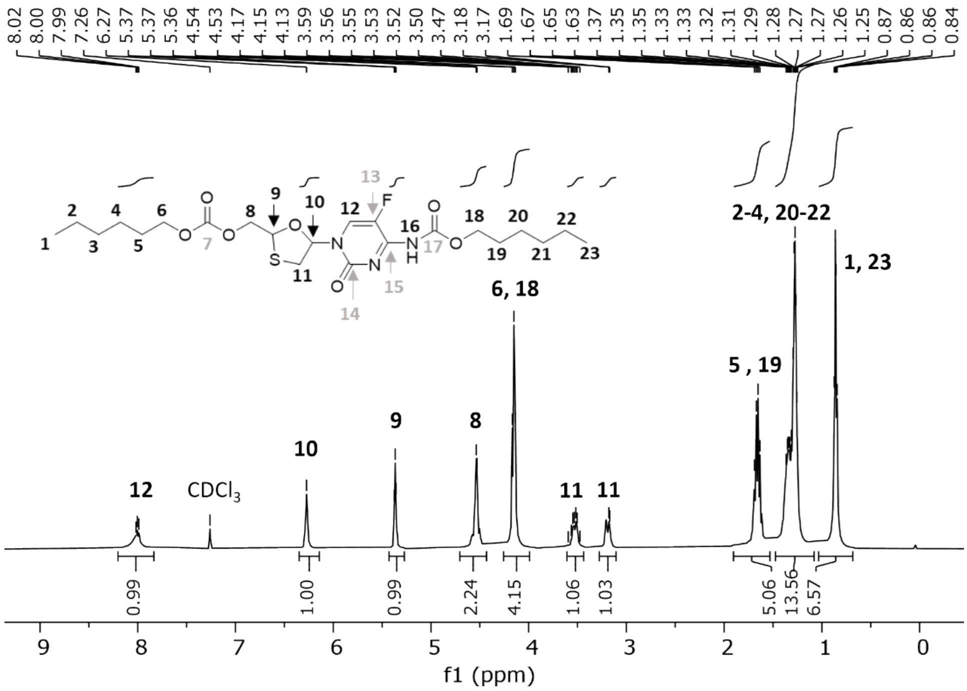

Figure S17. ^{1}H NMR (CDCl$_3$, 400 MHz) of disubstituted carbamate/carbonate FTC model compound ***5***.

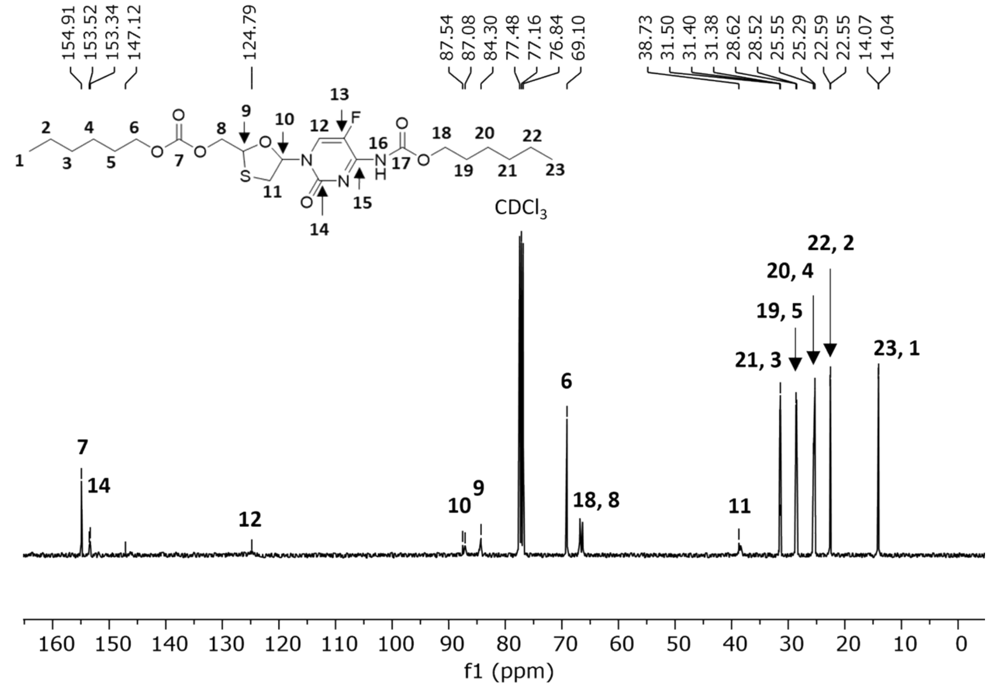

Figure S18. ^{13}C NMR (CDCl$_3$, 100 MHz) of disubstituted carbamate/carbonate FTC model compound ***5.***

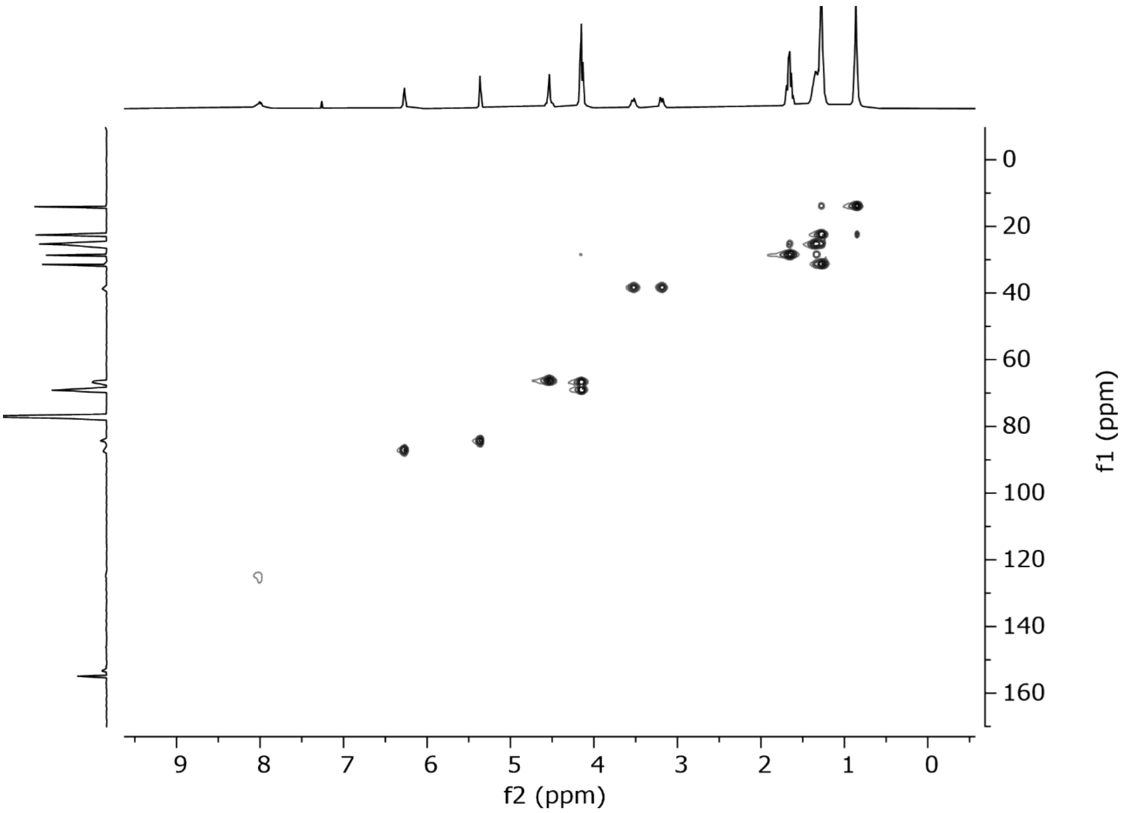

*Figure S19. HSQC ($CDCl_3$, 400 MHz) of disubstituted carbamate/carbonate FTC model compound **5**.*

Carothers equation modified:

$$p_c = \frac{2}{f_{av}} \qquad \text{Equation S1}$$

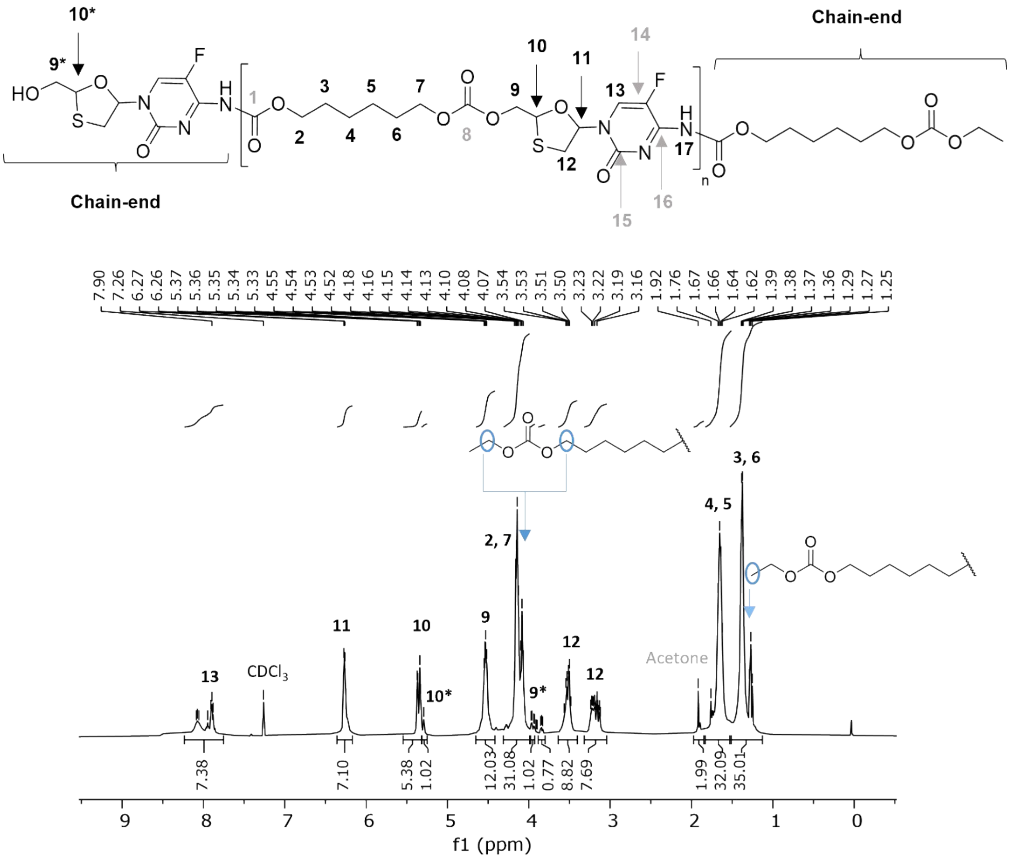

*Figure S20. ^{1}H NMR (CDCl$_3$, 400 MHz) of linear polymer poly(**hexyl**-FTC).*

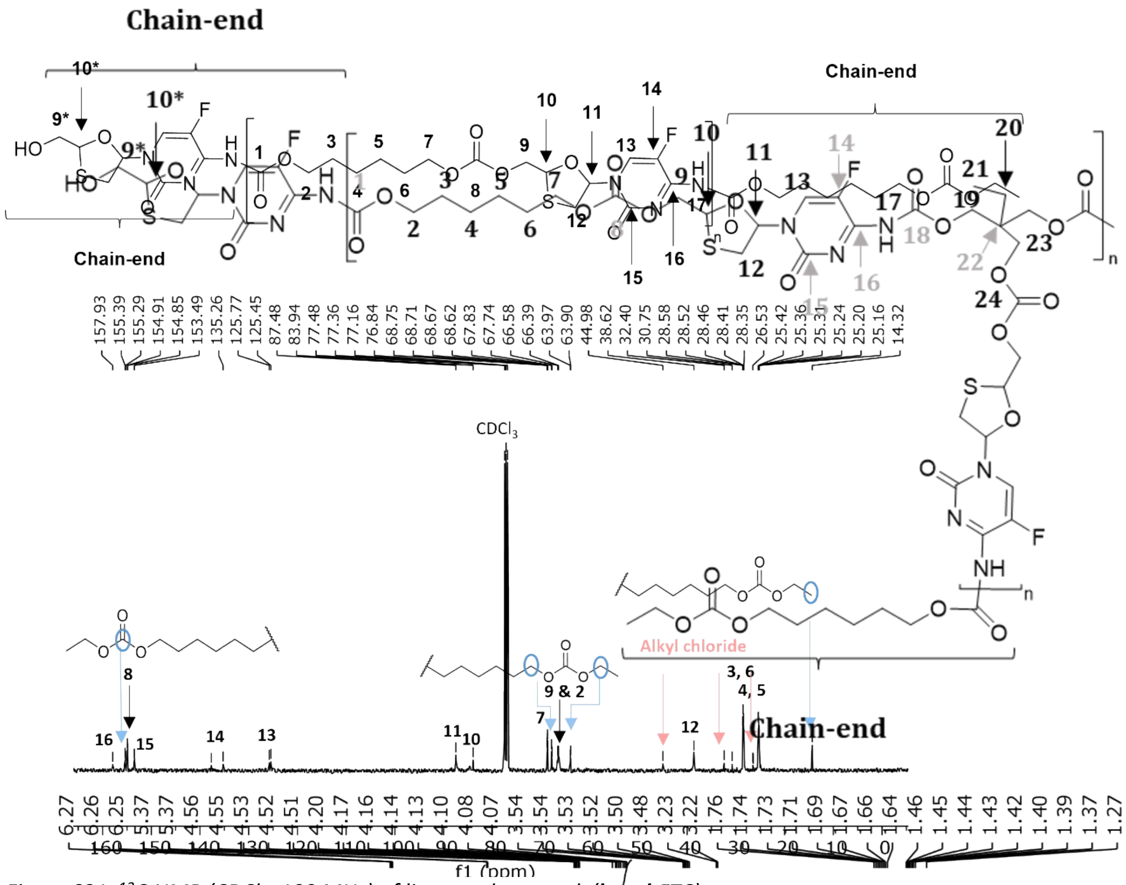

Figure S21. ^{13}C NMR ($CDCl_3$, 100 MHz) of linear polymer poly(**hexyl**-FTC).

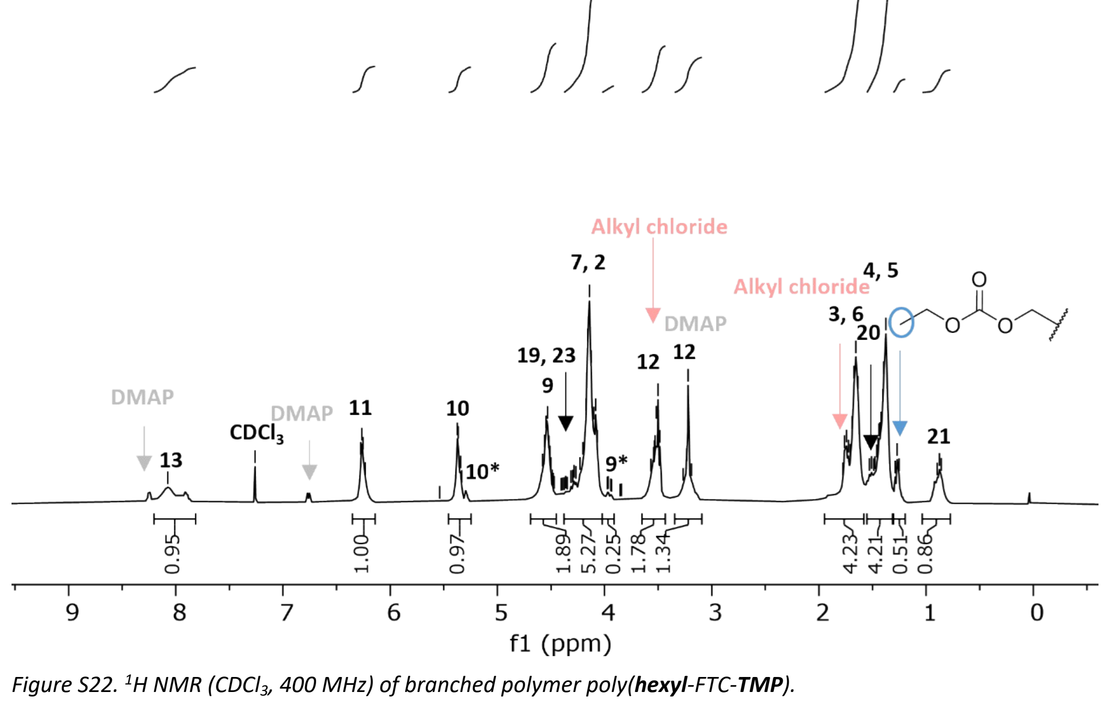

Figure S22. ^{1}H NMR ($CDCl_3$, 400 MHz) of branched polymer poly(**hexyl**-FTC-**TMP**).

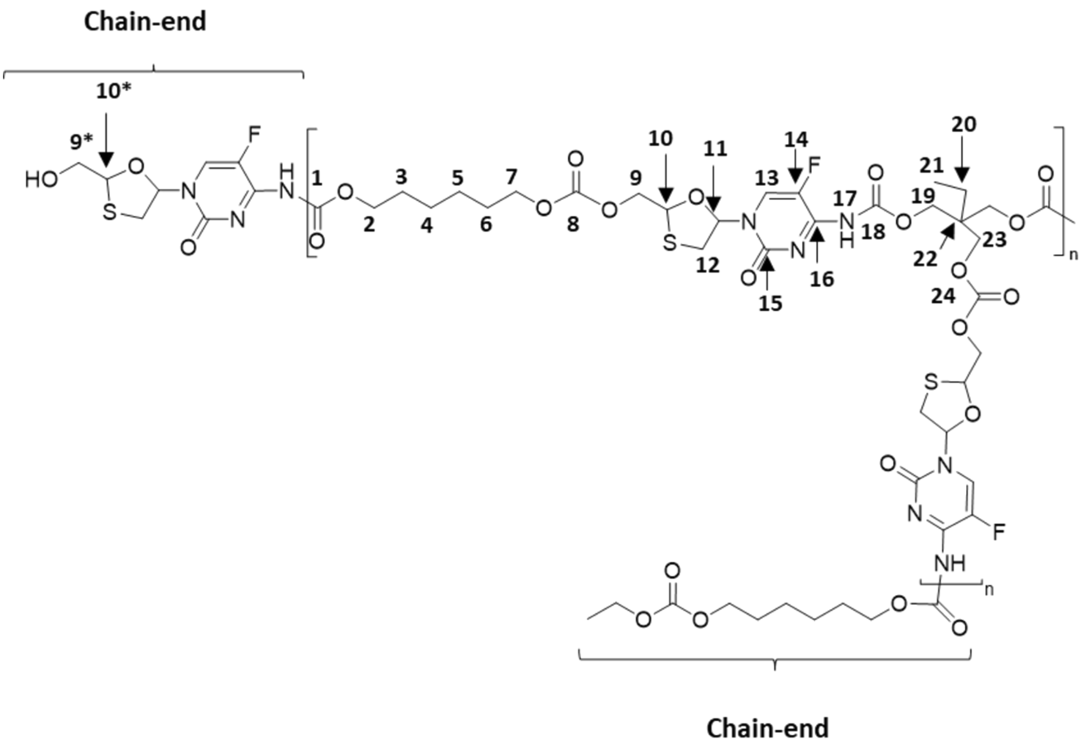

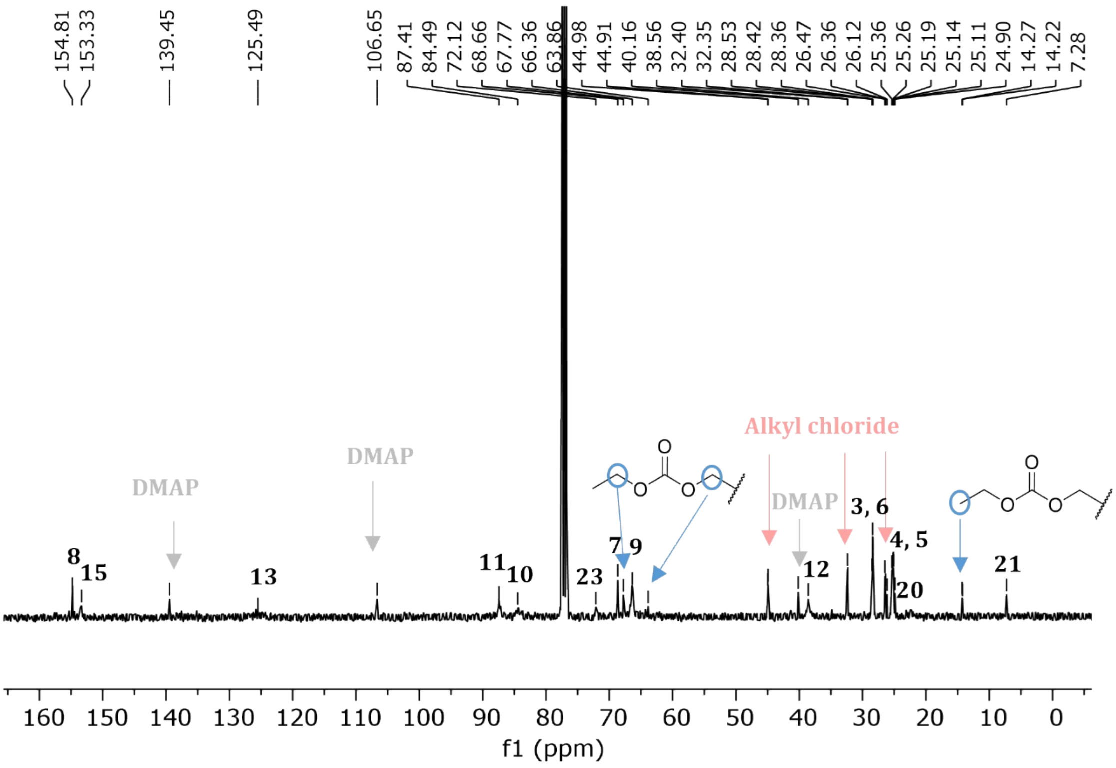

*Figure S23. ^{13}C NMR ($CDCl_3$, 100 MHz) of branched polymer poly(**hexyl**-FTC-**TMP**).*

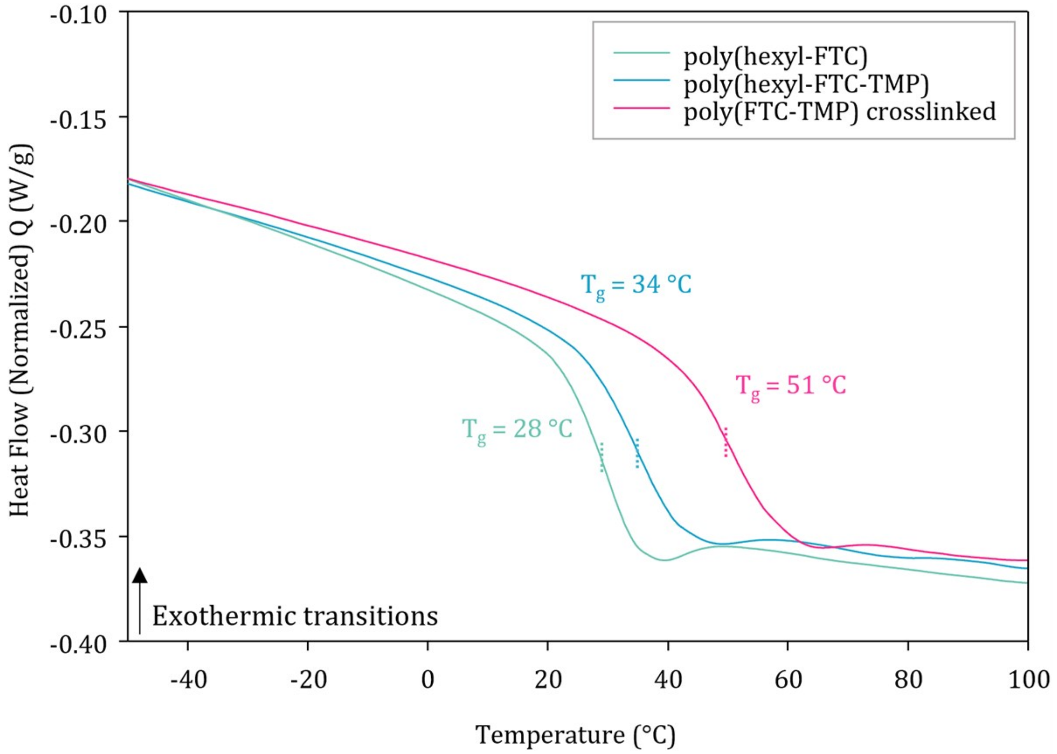

*Figure S24. Overlaid DSC thermograms of linear poly(**hexyl**-FTC) (green), branched polymer, poly(**hexyl**-FTC-**TMP**) (blue), and cross-linked polymer poly(FTC-**TMP**) (pink).*

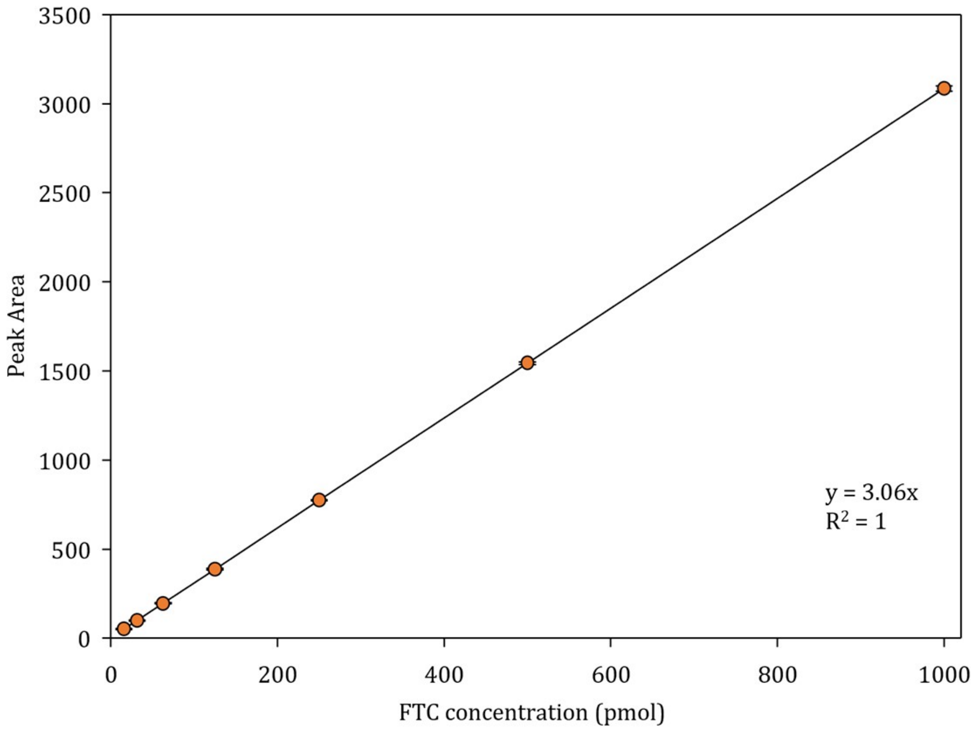

Figure S25. FTC standard concentration curve for HPLC release studies
